# Coastal Wetland Species *Rumex hydrolapathum*: Tolerance against Flooding, Salinity, and Heavy Metals for Its Potential Use in Phytoremediation and Environmental Restoration Technologies

**DOI:** 10.3390/life13071604

**Published:** 2023-07-21

**Authors:** Silvija Ieviņa, Andis Karlsons, Anita Osvalde, Una Andersone-Ozola, Gederts Ievinsh

**Affiliations:** 1 Department of Plant Physiology, Faculty of Biology, University of Latvia, 1 Jelgavas Str., LV-1004 Riga, Latviauna.andersone-ozola@lu.lv (U.A.-O.); 2 Institute of Biology, University of Latvia, 4 Ojāra Vācieša Str., LV-1004 Riga, Latvia; andis.karlsons@lu.lv (A.K.); anita.osvalde@lu.lv (A.O.)

**Keywords:** flooding, heavy metal tolerance, metal accumulation, phytoremediation, *Rumex hydrolapathum*, salinity tolerance, waterlogging

## Abstract

Plants with high biomass adapted to conditions of increased moisture and with significant salt tolerance appear to be particularly attractive candidates for phytoremediation studies. The aim of the present study was to examine the tolerance of *Rumex hydrolapathum* plants to freshwater, saltwater inundation, and soil contaminated with heavy metals, as well as its metal accumulation potential in controlled conditions. Six separate vegetation container experiments in controlled conditions were performed with *R. hydrolapathum* plants to study the effects of soil moisture, waterlogging with NaCl, soil Cd, soil Cr, soil Ni, and soil Pb in the form of a nitrate or acetate. Optimum plant growth occurred in waterlogged soil conditions. As the concentration of NaCl used for waterlogging increased, the mass of living leaves decreased, but that of dry leaves increased. As a result, the total biomass of leaves did not significantly change. *R. hydrolapathum* plants were extremely tolerant to Cd and Pb, moderately tolerant to Ni, and relatively sensitive to Cr. The plants had high capacity for metal accumulation in older and senescent leaves, especially for Na^+^, K^+^, Cd, and Ni. *R. hydrolapathum* plants can tolerate soil waterlogging with seawater-level salinity, which, together with the metal tolerance and potential for metal accumulation in leaves, make them excellently suited for use in a variety of wastewater treatment systems, including constructed wetlands.

## 1. Introduction

Environmental degradation as a result of soil salinization or accumulation of various pollutants, including heavy metals, has become a serious problem in many regions. An economically attractive and environmentally friendly solution to this problem is cultivation of highly tolerant wild plant species adapted to local conditions. In recent years, a lot of scientific information has accumulated about the use of plants in various phytoremediation systems to reduce the amount of harmful substances [1,2,3].

In order for plants to be used for such purposes, they must first be resistant to the relevant polluting factor. Second, to successfully perform phytoextraction, the plant must be able to absorb and accumulate the pollutant in the tissues of the aboveground parts. It is no less important to use species that have adapted to local conditions, so the identification of the characteristics of wild plants for the study of their phytoremediation properties is becoming more and more widespread. Salt-tolerant wild plants adapted to saline environments have emerged as effective candidates for use in environmental cleanup and restoration efforts [4]. The physiological properties that enable salt tolerance and resistance to heavy metals are similar or even overlapping. These include the existence of an efficient transport system, compartmentalization of metals in vacuoles or removal outside tissues, provision of osmotic balance, high efficiency of the enzymatic antioxidative system, and other properties [5].

Biomass accumulation potential is another plant feature to consider when looking for appropriate model species for phytoremediation studies. Many metallophytes and heavy metal accumulators are slow-growing plants and can be useful for studying of functional mechanisms. Particularly important is the combination of properties such as perennial type of growth, tolerance to soil waterlogging, high biomass accumulation rate, as well as the ability to accumulate metals. Such species can be used not only for soil remediation, but also to create various constructed wetlands for the treatment of polluted waters [6,7]. Thus, plants with high biomass adapted to conditions of increased moisture and with significant salt tolerance appear to be particularly attractive candidates for phytoremediation studies.

*Rumex hydrolapathum* Huds. (Polygonaceae) is the largest species of the genus, growing in marshes and on the banks of water bodies. There is very little research on the ecophysiology of *R. hydrolapathum*, but it is evident that the species can be found in soils with variable hydrological regimes and have a relatively high disturbance frequency [8]. However, it was indicated that *R. hydrolapathum* plants are found mostly in permanently flooded habitats, surviving by means of an aerenchymatous root system [9]. Therefore, *R. hydrolapathum* has been often classified as a helophyte species [10,11].

The taxonomically and ecologically closely related species *Rumex palustris* has been extensively used as a highly tolerant model plant for flooding and submergence studies [12]. In addition, several *Rumex* species have been characterized as metal tolerant accumulators, such as *Rumex hastatus* for Cu [13]; *Rumex crispus* for Cd, Pb, and Zn [14,15]; *Rumex obtusifolius* for Al [16]; and *Rumex nepalensis* for Pb and Zn [17,18]. In addition, *Rumex acetosa* has been described as a Cu-accumulating pseudo-metallophyte [19,20], and *Rumex acetosella* as a Cu-, Pb- and Zn-accumulating pseudo-metallophyte [21].

Previously, *R. hydrolapathum* has not been associated with salt-affected habitats, but it was recorded as a component of coastal grasslands in Finland [22]. In Sweden, *R. hydrolapathum* is characterized as only moderately salinity tolerant (indicator value 2 out of 5) [23]. Recently, we have shown that a *R. hydrolapathum* accession from a coastal wetland had high tolerance against biogenous heavy metals (Mn and Zn) [24] and that the accession had high tolerance against nitrate and nitrite as well as increased salinity tolerance [25]. It has been found that a general characteristic of *R. hydrolapathum* plants, which manifests in unfavorable conditions, is the ability to induce the aging process of older leaves and stimulate the formation of new leaves. However, *R. hydrolapathum* plants showed extreme sensitivity to increased ferrous iron concentrations among 44 fen plant species [26].

In order to ascertain the universal suitability of *R. hydrolapathum* accessions from salt-affected coastal wetlands for use in various phytoremediation technologies and environmental restoration measures, the aim of this study was to examine the plant’s tolerance to freshwater, saltwater inundation, and soil contaminated with non-biogenous heavy metals, as well as its metal accumulation potential in controlled conditions.

## 2. Materials and Methods

### 2.1. Plant Material, Cultivation, and Experimental Design

Seeds of *R. hydrolapathum* Huds. were collected in 2018 from plants growing in a sea-affected wetland habitat in Mērsrags, Latvia (Figure S1). The seeds were dried at room temperature and stored at 4 °C before use. For seed germination, they were placed on the surface of wet, autoclaved, soil-type, plant cultivation substrate Garden Soil (Biolan, Eura, Finland) in 1 L plant tissue culture containers and placed in a refrigerator for stratification at 4 °C in darkness. After 4 weeks, the containers were transferred to a growth cabinet with night/day temperatures of 15/20 °C, a 16 h photoperiod, and a photon flux density of photosynthetically active radiation of 50 μmol m^–2^ s^–1^. After the appearance of the second true leaf, the seedlings were individually transplanted to 200 mL plastic containers with Garden Soil, placed in 48 L plastic boxes, and gradually adjusted to greenhouse conditions.

The plants were cultivated in an experimental automated greenhouse (HortiMaX, Maasdijk, The Netherlands) with supplemental light provided by Master SON-TPIA Green Power CG T 400W (Philips, Amsterdam, The Netherlands) and Powerstar HQI-BT 400 W/D PRO (Osram, Munich, Germany) lamps (photon flux density of photosynthetically active radiation of 380 μmol m^–2^ s^–1^ at the plant level), with a 16 h photoperiod. The air relative humidity was 60–70%, and the day/night temperatures were 23/16 °C.

Six separate vegetation container experiments in controlled conditions were performed with *R. hydrolapathum* plants at the stage of 4–5 true leaves (Table 1). Five individual plants per treatment were used. When the height of the seedlings reached 5–10 cm, they were transplanted to 1.2 L plastic containers filled with a 1 L mixture of quartz sand (Saulkalne S, Saulkalne, Latvia) and Garden Soil (Biolan, Eura, Finland) in a 1:3 (*v*/*v*) ratio. The exception was plants for the Ni experiment, where the appropriate amount of the respective salt was added to the substrate before the final transplanting. For other experiments, the treatments were started one week later. Every second week, the plants were fertilized with Yara Tera Kristalon Green fertilizer (Yara International, Oslo, Norway), 0.5 g L^–1^ (50 mL per plant). However, for the soil moisture and NaCl waterlogging experiments, the final fertilization was performed before the start of the treatments.

For the soil moisture experiment, four levels of moisture were maintained (Table 1). Substrate water level and electrical conductivity (EC) was monitored daily with an HH2 moisture meter equipped with a WET-2 sensor (Delta-T Devices, Burwell, UK). The WET-2 sensor uses the principle of frequency domain reflectometry to measure dielectric properties of the substrate, which are converted to water content (% vol) by the instrument using calibration curves. For the low moisture treatment, the substrate water content was maintained at 25–35% and was designated as 30% treatment. For the moderate moisture treatment, the substrate water content was maintained at 40–60% and was designated as 50% treatment. For the soil waterlogging treatment, containers with plants were placed inside 4 L containers and the water level inside the outer container was maintained up to half the height of the inner container, and was designated as 80% treatment. For the soil flooding treatment, the containers with plants were placed inside 4 L containers and the water level inside the outer container was maintained about 3 cm above the soil level, and designated as 85% treatment. Deionized water was used, and its volume was completely changed every week. At that point, some of the plants were treated with 200 mM NaCl, 100 mL per plant, for three consecutive weeks.

For the NaCl waterlogging experiment, all plant containers were placed inside 4 L containers and the water level inside the outer container was maintained up to half the height of the inner container. For control plants, deionized water was used, but for the other treatments, the concentration of NaCl in water was increased stepwise within four weeks to 25, 50, 100, 200, 400 mM.

For heavy metal treatments except Ni, plants were treated with water-solubilized salts in increasing concentration within three weeks until the required concentration in the substrate was reached. Substrate moisture level was monitored daily and maintained at 60–70%. Plants were cultivated for 6–7 weeks after the start of the treatments.

### 2.2. Physiological Measurements

Physiological status of plants was evaluated by non-destructive measurement of photosynthesis-related parameters, leaf chlorophyll concentration and chlorophyll *a* fluorescence analysis, weekly during plant cultivation. For measurements, photosynthetically most important leaf of each plant was selected. Preliminary tests using chlorophyll *a* fluorescence parameter Performance Index Total showed that it was the longest leaf of the plant at that particular moment. For each plant, two measurements of each chlorophyll concentration and chlorophyll *a* fluorescence were performed, with a total of 10 measurements per treatment.

Chlorophyll concentration in plant leaves was measured by a chlorophyll meter CCM-300 (Opti-Sciences, Hudson, NH, USA). Chlorophyll a fluorescence was measured in leaves that had been dark adapted for at least 20 min by a Handy PEA fluorometer (Hansatech Instruments, King’s Lynn, UK). For the characterization of photochemical activity, the chlorophyll *a* fluorescence parameter Performance Index Total was used. This parameter is a complex indicator of photochemical efficiency combining three function-related (trapping of an absorbed exciton, electron transport between the photosystems, reduction of end-electron acceptors) and structure-related (antenna chlorophyll per reaction center chlorophyll) parameters [27].

### 2.3. Termination of the Experiments and Measurements

Plant leaves were detached and separated into different age/developmental groups (dead dry, small, large, and new). If roots had formed outside the substrate in the water of the outer container or individual tray, they were collected separately. Roots were carefully washed to remove any attached substrate particles and separated into fine roots and large roots. All plant parts were weighed separately before and after drying in an oven at 60 °C until constant mass. Tissue water content was calculated based on the dry mass.

Concentrations of Na^+^ and K^+^ as well as EC were measured in tissue water extracts in five biological replicates (samples from individual plants). Plant tissues were crushed by hand and a homogeneous sample (0.2 g) was ground to a fine powder with a mortar and pestle. Then, 10 mL of deionized water was added and stirred with the pestle for 1 min. After filtration through nylon mesh cloth (No. 80), the homogenate was used for measurement of ion concentration by LAQUAtwin compact meters B-722 (Na^+^) and B-731 (K^+^), and EC by a LAQUAtwin conductivity meter B-771 (Horiba, Kyoto, Japan). At least three analytical replicates were performed for each sample and the average value was calculated.

Concentrations of Cd, Cr, Ni and Pb in different plant parts were measured in triplicate. About 2 g of plant material was fixed 2–3 min at 105 °C, then dried at 60 °C to constant mass and ground. Material was dry-ashed with HNO_3_ vapor and re-dissolved in a 3% HCl solution. Determination of heavy metals was performed by microwave plasma atomic emission spectrometry (4200 MP-AES, Agilent, Santa Clara, CA, USA) according to the manufacturer’s instructions.

### 2.4. Data Analysis

Results were analyzed by KaleidaGraph (v. 5.0, Synergy Software, Reading, PA, USA). Statistical significance of differences was evaluated by one-way ANOVA using post hoc analysis with a minimum significant difference. Significant differences were indicated by *p* < 0.05.

## 3. Results

### 3.1. Effect of Soil Moisture and NaCl

In the soil moisture experiment, regular measurements of moisture and electrical conductivity were made during *R. hydrolapathum* cultivation. Time-varying moisture levels were characteristic for plants maintained in low and medium moisture conditions, but were relatively stable in plants in waterlogged and flooded soils (Figure S2A). A gradual increase in NaCl concentration resulted in a corresponding rise in soil EC, but it was less pronounced for low and medium moisture conditions (Figure S2B). Epinastic leaf growth was a characteristic feature of plants cultivated in both waterlogged and flooded soil (Figure S3).

**Figure 1:**
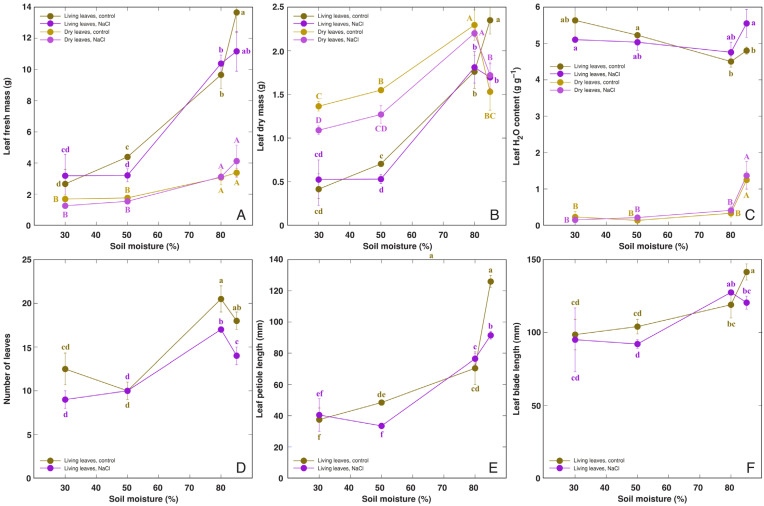
Effect of soil moisture and NaCl treatment on fresh mass of dry and living leaves (**A**), dry mass of dry and living leaves (**B**), water content in leaves (**C**), number of leaves (**D**), leaf petiole length (**E**), and leaf blade length (**F**) of *Rumex hydrolapathum* plants. Results represent mean values from five replicates ± SE. Different letters of the same color and the case indicate statistically significant differences (*p* < 0.05).

The morphological parameters clearly indicated that the optimum growth of *R. hydrolapathum* plants was attained in waterlogged soil conditions, but further soil flooding only had negligible effects (Figure 1). The length of leaf blades tended to increase in waterlogged and flooded conditions (Figure 1F), but increases in leaf petiole length were more pronounced (Figure 1E). Treatment with a low dose of NaCl had a significant negative effect on the dry mass of living leaves in low and medium moisture conditions (Figure 1B), but the number of leaves significantly decreased in waterlogged and flooded conditions (Figure 1D). In addition, the flooding-stimulated increase in leaf length was suppressed by NaCl treatment.

**Figure 2:**
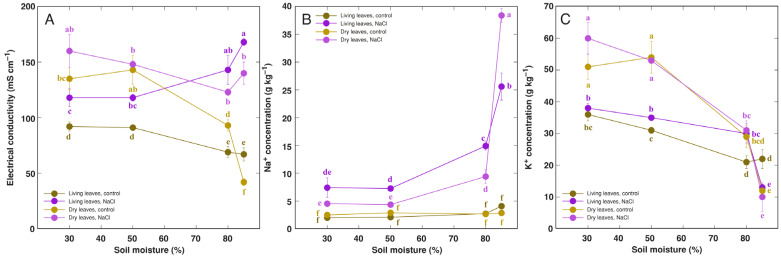
Effect of soil moisture and NaCl treatment on electrical conductivity (**A**), Na^+^ concentration (**B**), and K^+^ concentration (**C**) in leaf extracts of *Rumex hydrolapathum* plants. Results represent mean values from five replicates ± SE. Different letters of the same color indicate statistically significant differences (*p* < 0.05).

Soil waterlogging and flooding resulted in a decrease in soluble ion concentration in leaf tissues of control plants, as evidenced by respective changes in tissue EC levels (Figure 2A). These changes were associated with a decrease in tissue K^+^ concentration (Figure 2C). Plants treated with NaCl accumulated Na^+^ in leaves, and the accumulation potential was more pronounced in waterlogged and, especially, flooded conditions (Figure 2B), leading to corresponding changes in EC values (Figure 2A). K^+^ concentration was not affected by NaCl treatment (Figure 2C).

Leaf chlorophyll concentration was negatively affected by soil flooding at the early stages after the start of the treatment and by flooding throughout the experiment, in comparison to dry-soil-grown plants (Figure S4A). Treatment with NaCl had a minor negative effect only in conditions of soil waterlogging (Figure S4B). The chlorophyll *a* fluorescence parameter Performance Index was significantly increased in plants grown with increased moisture, but the effect was stable only for waterlogged plants (Figure S4C). Treatment with NaCl decreased the Performance Index only in low and medium moisture conditions (Figure S4D).

### 3.2. Effect of Soil Waterlogging with NaCl

When *R. hydrolapathum* plants were cultivated in waterlogged soil caused by using increasing concentrations of NaCl, both soil EC and Na^+^ concentration increased, but the effect was not linearly dependent on the NaCl concentration used (Figure S5). These experimental conditions, associated with a certain degree of nutrient deprivation due to absence of additional fertilization in waterlogged conditions, resulted in relatively high rate of leaf senescence during plant cultivation (Figure S5). Therefore, the number and biomass of leaves were registered separately for dry and living leaves.

**Figure 3:**
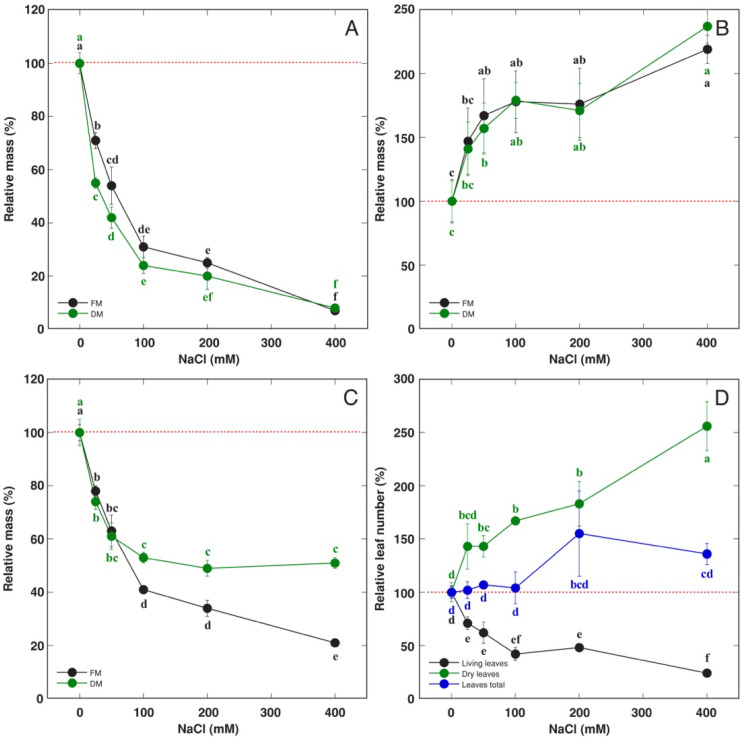
Effect of soil waterlogging with increasing concentration of NaCl on relative mass of living leaves (**A**), relative mass of dry leaves (**B**), relative mass of all leaves (**C**), relative leaf number (**D**) of *Rumex hydrolapathum* plants. Results represent mean values from five replicates ± SE. Different letters of the same color indicate statistically significant differences (*p* < 0.05). The red dotted line indicates control level.

**Figure 4:**
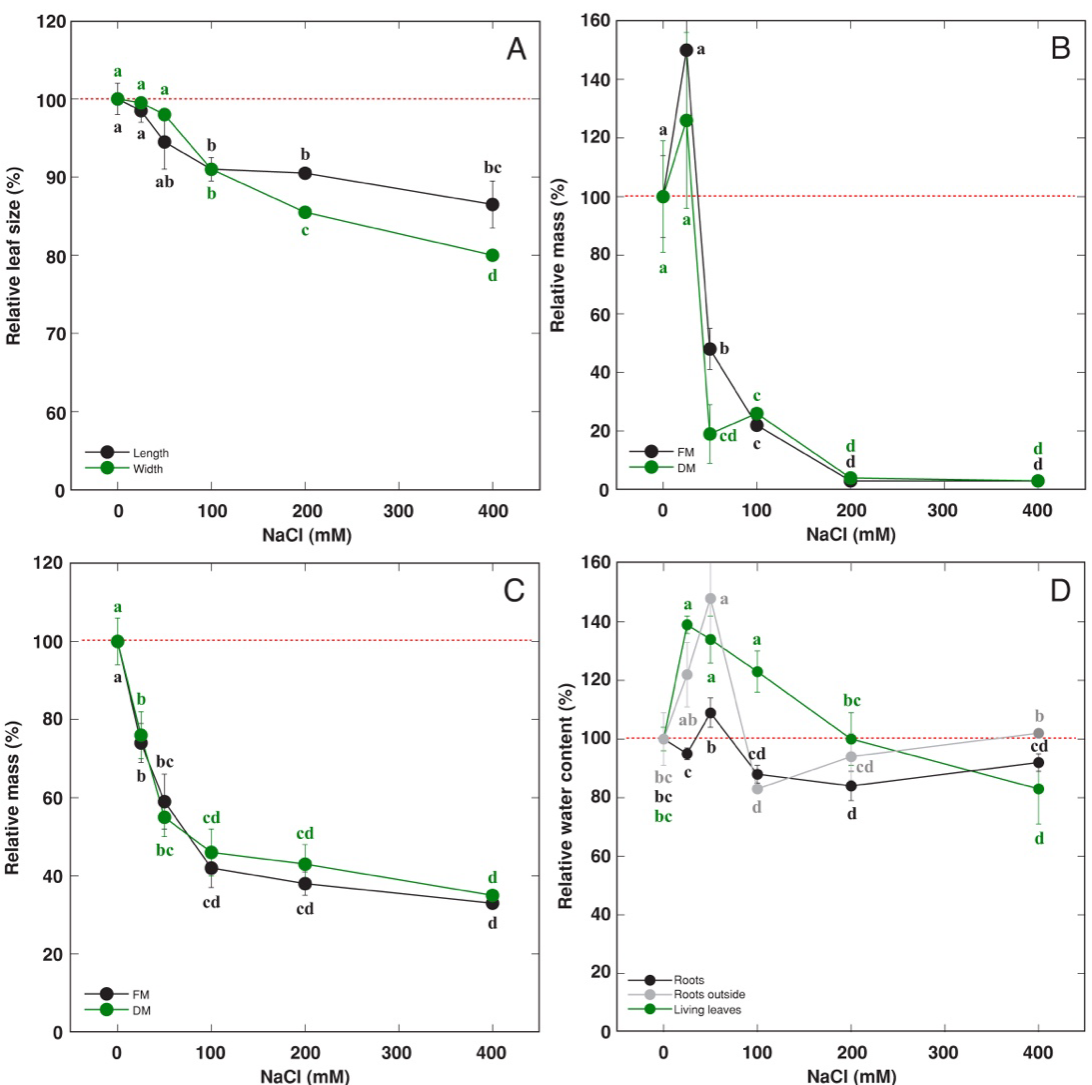
Effect of soil waterlogging with increasing concentration of NaCl on relative size of living leaves (**A**), relative mass of roots outside the container (**B**), relative mass of roots inside the container (**C**), relative water content in different parts (**D**) of *Rumex hydrolapathum* plants. Results represent mean values from five replicates ± SE. Different letters of the same color indicate statistically significant differences (*p* < 0.05). The red dotted line indicates control level.

Both the fresh and dry mass of living leaves decreased with increasing concentration of NaCl used for waterlogging (Figure 3A), but the mass of dry leaves increased (Figure 3B). As a result, the total biomass of leaves did not significantly change (Figure 3C). Also, number of living leaves decreased but that of dry leaves decreased along with increasing NaCl concentration, and the total number of leaves did not change significantly (Figure 3D). The size of living leaves decreased with increasing NaCl concentration (Figure 4A). Waterlogging conditions stimulated root development and elongation in the water in the outer container outside of the soil, and this effect was strongly inhibited by a high NaCl concentration in that water (Figure 4B). The mass of roots in soil also was negatively affected by NaCl, but the response saturated at 100 mM (Figure 4C). The water content significantly increased in both living leaves and roots outside soil at 25–100 and 50 mM NaCl, respectively, with no significant changes in roots located in soil (Figure 4D).

**Figure 5:**
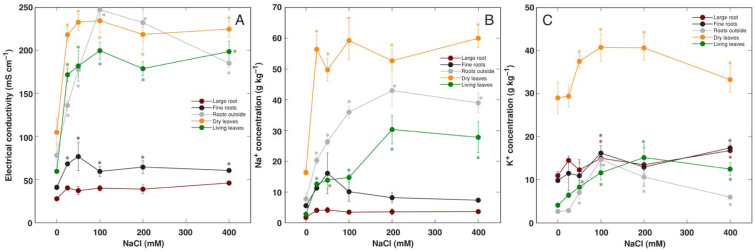
Effect of soil waterlogging with increasing concentration of NaCl on electrical conductivity (**A**), Na^+^ concentration (**B**), K^+^ concentration (**C**) in different parts of *Rumex hydrolapathum* plants. Results represent mean values from five replicates ± SE. Asterisks of the same color indicate statistically significant differences (*p* < 0.05) from control.

The plants treated with NaCl accumulated high concentrations of soluble ions in their leaves and roots outside the container as reflected by an increased EC (Figure 5A). The increase in EC was relatively small in fine roots and no increase was seen in large roots. Accumulation of Na^+^ as a result of increasing NaCl concentration was more pronounced in dry leaves, followed by roots outside the container and living leaves (Figure 5B). The accumulation potential of K^+^ in dry leaves was also high and significantly increased in NaCl-treated plants (Figure 5C). The leaf chlorophyll concentration was only relatively slightly increased by NaCl treatment at the later stages of plant cultivation (Figure S7A), and the chlorophyll *a* fluorescence parameter Performance Index tended to increase at 100–400 mM NaCl at the later stages (Figure S7B).

### 3.3. Effect of Heavy Metals: Cd

**Figure 6:**
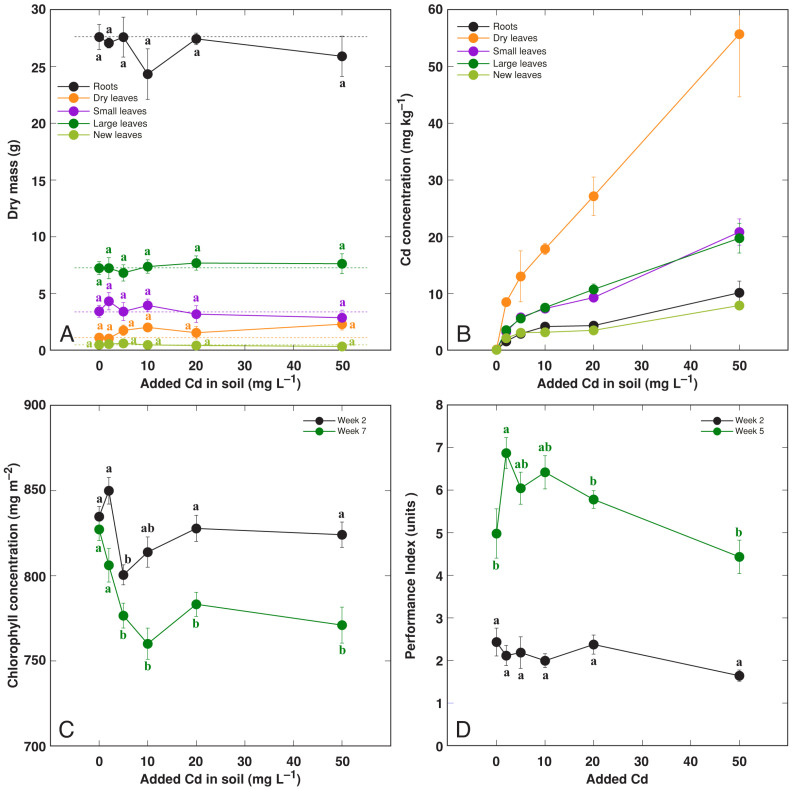
Effect of treatment with increasing concentration of Cd on dry mass of different parts (**A**), Cd concentration in different parts (**B**), leaf chlorophyll concentration (**C**), leaf Performance Index (**D**) of *Rumex hydrolapathum* plants. For (**A**,**B**), results represent mean values from five replicates ± SE. For (**B**,**C**), results represent mean values from 10 independent measurements for each point ± SE. Different letters of the same color indicate statistically significant differences (*p* < 0.05). The dotted line indicates control level.

The morphology of Cd-treated *R. hydrolapathum* plants did not change with increasing concentration in the soil treatment (Figure S8). The biomass of any part of the plant was not significantly affected by the treatment (Figure 6A). The highest Cd accumulation potential was in dry leaves, followed by large leaves and small leaves, and it was the lowest in roots and new leaves (Figure 6B). The leaf chlorophyll concentration decreased in Cd-treated plants at the later stages of cultivation (Figure 6C), but the chlorophyll *a* fluorescence parameter Performance Index tended to increase at low Cd levels at the later stages of cultivation (Figure 6D).

### 3.4. Effect of Heavy Metals: Cr

**Figure 7:**
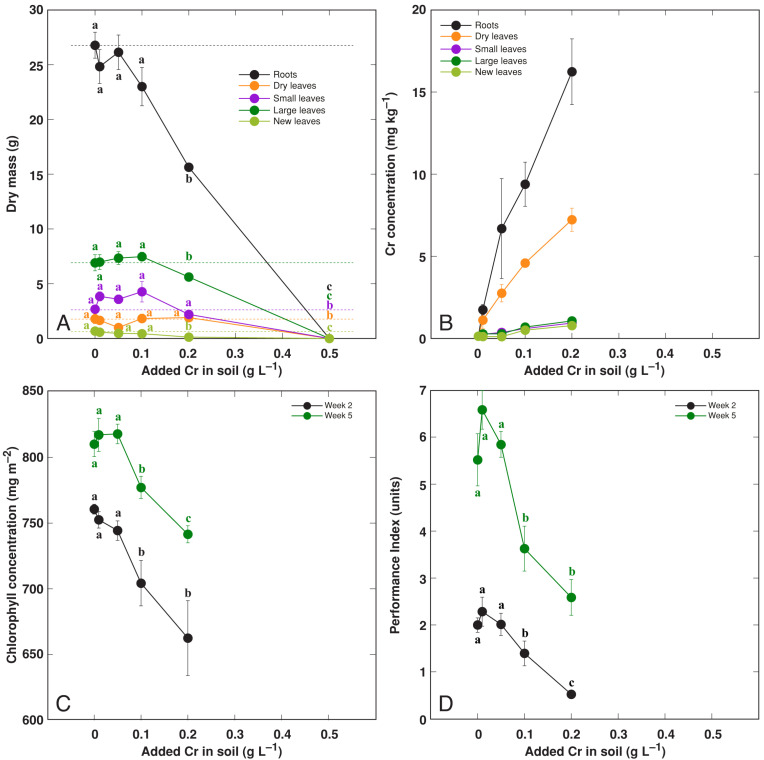
Effect of treatment with increasing concentration of Cr on dry mass of different parts (**A**), Cr concentration in different parts (**B**), leaf chlorophyll concentration (**C**), leaf Performance Index (**D**) of *Rumex hydrolapathum* plants. For (**A**,**B**), results represent mean values from five replicates ± SE. For (**B**,**C**), results represent mean values from 10 independent measurements for each point ± SE. Different letters of the same color indicate statistically significant differences (*p* < 0.05). The dotted line indicates control level.

Plant treatment with Cr resulted in relatively severe effects on the growth of *R. hydrolapathum* plants: at the highest Cr concentration (500 mg L^–1^), all plants died three weeks after the full treatment (Figure S9), and plant growth was significantly inhibited at 200 mg L^–1^ Cd (Figure 7A). The majority of Cr accumulated in roots followed by dry leaves and was very low in living leaves (Figure 7B). Increasing soil Cr concentration resulted in a near-linear decrease in leaf chlorophyll concentration (Figure 7C) and the chlorophyll *a* fluorescence parameter Performance Index (Figure 7D).

### 3.5. Effect of Heavy Metals: Ni

**Figure 8:**
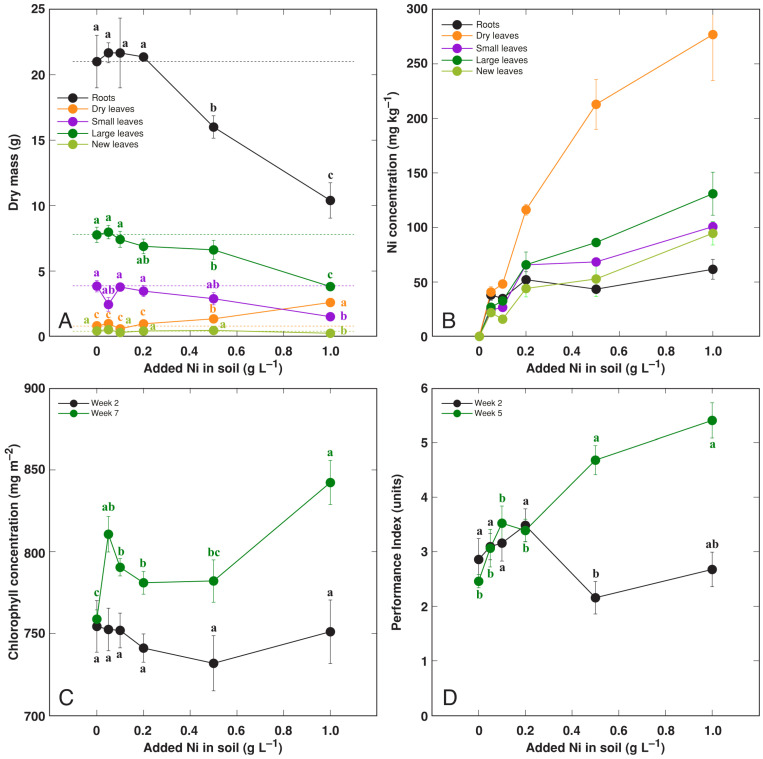
Effect of treatment with increasing concentration of Ni on dry mass of different parts (**A**), Ni concentration in different parts (**B**), leaf chlorophyll concentration (**C**), leaf Performance Index (**D**) of *Rumex hydrolapathum* plants. For (**A**,**B**), results represent mean values from five replicates ± SE. For (**B**,**C**), results represent mean values from 10 independent measurements for each point ± SE. Different letters of the same color indicate statistically significant differences (*p* < 0.05). The dotted line indicates control level.

*R. hydrolapathum* plants cultivated in Ni-containing soils showed decreased growth with increasing metal concentration (Figure S10 and Figure 8A). The biomass of roots, new leaves, and small leaves was most negatively affected. Ni accumulation potential was high in dry leaves, significantly lower in living leaves, and low in roots (Figure 8B). Both leaf chlorophyll concentration (Figure 8C) and Performance Index (Figure 8D) increased in the later stages of cultivation in plants treated with a high concentration of Ni.

### 3.6. Effect of Heavy Metals: Pb in the Form of Nitrate and Acetate

**Figure 9:**
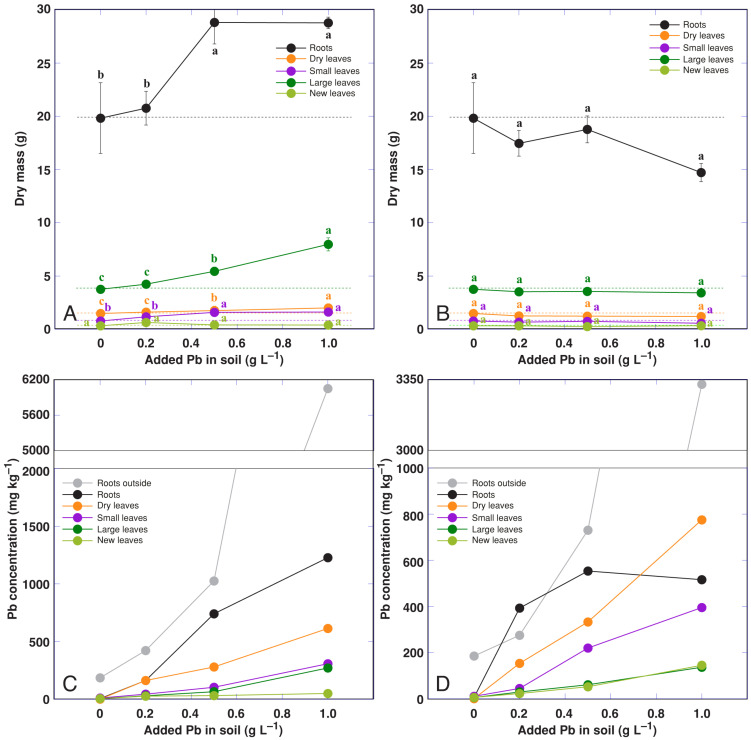
Effect of increasing concentration of Pb in the form of a nitrate on dry mass of parts (**A**) and Pb accumulation in different parts (**C**), and Pb in the form of an acetate on dry mass of parts (**B**) and Pb accumulation in different parts (**D**) of *Rumex hydrolapathum* plants. Results represent mean values from five replicates ± SE. Different letters of the same color indicate statistically significant differences (*p* < 0.05). The dotted line indicates control level.

In order to separate the effect of Pb from that of nitrate, *R. hydrolapathum* plants were subjected to treatment with Pb nitrate or Pb acetate at identical Pb concentrations (Figure S11). Treatment with Pb acetate resulted in a significant increase in soil pH (Figure S12). The biomass of roots, large leaves, and dry leaves significantly increased in plants treated with 500 and 1000 mg Pb in the form of a nitrate (Figure 9A), but no effect on growth was evident for plants treated with Pb acetate (Figure 9B). The accumulation of Pb was relatively more pronounced in plants treated with Pb nitrate (Figure 9C) in comparison to that for plants treated with Pb acetate (Figure 9D). The maximum Pb accumulation potential was seen in roots outside the soil in both treatment types (Figure 9C,D). It was also high in the roots of nitrate-treated plants and was lower in dry leaves, with the lowest level in living leaves. Treatment with Pb nitrate (500 and 1000 mg L^–1^) and Pb acetate (100 mg L^–1^) resulted in increased leaf chlorophyll concentration at the early stages of the experiment (Figure S13A), but some decrease was evident for Pb acetate-treated plants at the later stages (Figure S13B). The highest concentration of Pb nitrate also increased the Performance Index at the early stages of cultivation (Figure S13C), but no effect was evident at the later stages (Figure S13D).

## 4. Discussion

### 4.1. Abiotic Stress Tolerance

It is usually expected that plant species native to permanently wet habitats will have higher tolerance against soil waterlogging than soil drying when cultivated in controlled conditions. For example, *Saussurea esthonica*, a rare species of Compositae found only in wet inland alkaline fen habitats, showed the most intense growth in waterlogged soil [28]. Also, in natural conditions of dune slacks, experimentally assessed tolerance of waterlogging and submergence for different species results in particular zonation patterns of these species [29]. In the present study, *R. hydrolapathum* plants from a coastal wetland showed optimum growth in waterlogged soil (Figure 1). The rise of the water level above the soil level (flooding) had a relatively small effect on plant growth and morphology in comparison to plants in waterlogged conditions. A significant increase in petiole (Figure 1E) and leaf blade (Figure 1F) length, as well as hyponastic leaf growth were among the most typical responses to flooding. As there were only small differences in soil water content between waterlogged and flooded treatments (Figure S2A), it is logical to ask how the plants perceived the difference between these two states, and was the soil saturated with water in both cases? As oxygen deprivation in waterlogged soil is a significant physiologically factor, it seems that the surface soil oversaturated with water still acts as a supplier of oxygen for roots that intensively settle near the surface in conditions of excessive moisture. In flooded conditions, even if the water level is only several centimeters above the soil surface, no oxygen can reach the roots directly. In fully submerged conditions, the accumulation of ethylene in photosynthesizing tissues due to low gas diffusion in water is considered to be a major factor regulating leaf hyponastic growth and petiole elongation [12]. In contrast, root hypoxia-induced signaling seems to be the main initial control point for soil waterlogging-induced responses [30].

The current accession of *R. hydrolapathum* is exposed to fluctuating salinity in natural conditions due to periodic soil waterlogging with seawater [31]. Therefore, the effect of salinity on plant growth in the present study was tested in waterlogged conditions. It is well-established that the soil moisture level affects plant salinity tolerance [32]. Usually, flooding with saline water has more severe consequences for plant growth in comparison to these occurring in moderately moist soil. However, it can be expected that plant species adapted to inundation with seawater will not show such differences.

In moderate salinity conditions, salinity had an inhibitory effect on leaf growth of *R. hydrolapathum* plants only in low and medium soil moisture conditions, but not in waterlogged soil (Figure 1E). However, with increasing NaCl concentration in the water used for soil waterlogging, the number and biomass of living leaves progressively decreased but those of dry leaves increased (Figure 3).

Soil waterlogging-induced formation of adventitious roots with increased porosity is a characteristic response in wetland-adapted *Rumex* species, including *R. hydrolapathum*. Interestingly, waterlogging resulted in root formation outside the soil in the water of the outer container, and this was suppressed by increasing NaCl concentration. However, these roots had extreme potential for accumulation of both Na ions (Figure 5B) and Pb (Figure 9C,D).

The results of the experiments on the effect of moisture regimes and waterlogging with NaCl could have been influenced by the fact that no additional fertilization was applied from the beginning of the implementation of the treatment regimes. In the first experiment, because the amount of deionized water used to treat the plants varied, the use of a nutrient solution could lead to differences in mineral availability between the treatments. For sake of comparison, identical conditions were used in the second experiment. Moreover, a similar situation appears during soil waterlogging in natural conditions of seawater-affected coastal habitats [31]. Consequently, *R. hydrolapathum* plants were under nutrient limited conditions, which appeared as an increased rate of senescence of older leaves and the formation of new leaves as a result of resource remobilization.

The physiological status of photosynthetically active leaves remained relatively stable in *R. hydrolapathum* plants subjected to various soil moisture levels as well as with waterlogging with NaCl, as indicated by only small changes in photosynthesis-related parameters. Moreover, the Performance Index was higher for plants grown in waterlogged and flooded conditions (Figure S4C). It has been shown previously that the chlorophyll *a* fluorescence parameter Performance Index Total is an extremely sensitive indicator for suboptimal changes in environmental conditions [33]. With respect to soil moisture, for alkaline fen species *Saussurea esthonica* plants with optimum growth in high soil moisture conditions, increased substrate moisture resulted in a significantly increased Performance Index [28]. Usually chlorophyll *a* fluorescence parameters decrease in salt-sensitive plants as a result of NaCl treatment, as shown for *Eruca sativa* [34], *Boehmeria nivea* [35], and *Medicago truncatula* [36]. However, such a negative effect does not appear in halophyte species [37,38,39].

### 4.2. Tolerance against Heavy Metals

Plant responses to heavy metals at different levels of functional organization as well as mechanisms of tolerance have been subjects of extensive studies in previous decades. Different physiological aspects of the effects of heavy metals have been reviewed recently, including those for Cd [40], Cr [41], Ni [42], and Pb [43], and readers are requested to refer to these sources for further information.

*R. hydrolapathum* plants were extremely tolerant to Cd and Pb, moderately tolerant to Ni, and relatively sensitive to Cr. Previously, it was shown that *R. hydrolapathum* plants exhibited high tolerance to the biogenous heavy metals Mn and Zn, as the leaf dry mass of plants was not negatively affected by up to 1 g Zn or Mn per L of soil [24]. Other *Rumex* species have been tested for their heavy metal tolerance.

The different effects in the case of Pb nitrate (growth stimulation) and Pb acetate (no effect on growth) reconfirmed the nitrophilous characteristic of *R. hydrolapathum*. Similar effects of Pb nitrate and Pb acetate were described for another semiaquatic plant species from a salt-affected coastal beach, *Ranunculus sceleratus* [44]. Previously, it was shown that Na and K salinity in a form of nitrates showed positive effects on the growth of *R. hydrolapathum* plants [25].

The majority of crop species are relatively sensitive to increased soil Cr level. Thus, a large sensitivity has been exhibited by crops such as *Avena sativa*, where treatment with 150 mg kg^–1^ Cr(VI) resulted in almost complete growth depression of the plants [45]. However, *Triticum aestivum* plants only showed a shoot biomass reduction of 75% at 200 mg kg^–1^ Cr(VI) [46]. In the present study, there was a 21% and 41% reduction in leaf and root growth, respectively, at 200 mg L^–1^ Cr, and no plants survived at the 400 mg L^–1^ Cr concentration (Figure 7).

Resistance vs. susceptibility to specific metals by *R. hydrolapathum* plants was reflected by respective changes in photosynthesis-related parameters, leaf chlorophyll concentration, as well as chlorophyll *a* fluorescence. The metal treatments without negative effects on plant growth did not result in a decrease in photosynthesis-related parameters, but treatment with Cr decreased both leaf chlorophyll concentration and the Performance Index in a concentration-dependent manner (Figure 7C,D). The negative effects of Cr treatment on photosynthesis have been reported previously [47].

### 4.3. Metal Accumulation Potential

In contrast to metal accumulating species which have a high potential for metal transport to aboveground parts leading to efficient tissue or cellular level sequestration, the majority of herbaceous plants accumulate metals exclusively in root tissues irrespective of their tolerance to the particular metal. Metal-excluding species are found in both heavy metal-tolerant plants and among halophytes.

In another study with *R. hydrolapathum*, the plants had a pronounced ability to accumulate the heavy metals Zn and Ni in their roots, but their translocation capacity to shoots was low [48]. In the present study, more Cd and Ni accumulated in leaves (especially, in dry leaves) than in roots, but more Cr and Pb accumulated in the roots of *R. hydrolapathum* plants.

The shoot accumulation potential for Cd only reached 2 mg kg^–1^ for the pseudo-metallophyte species *Rumex acetosa* [19]. In *Rumex acetosa* from different sites with soil Cd concentrations of 0.05–15.15 mg kg^–1^, the Cd concentration in leaves ranged from 0.04 to 0.96 mg kg^–1^ FM [49]. When *Rumex crispus* plants were grown on mine tailings, nearly equal concentrations of Cd were accumulated in leaves (37–44 mg kg^–1^) and roots (33–38 mg kg^–1^) [15]. The hyperaccumulation threshold concentration for Cd has been set at 100 mg kg^–1^ [50]. Consequently, with Cd concentration reaching 56 and 20 mg kg^–1^ in dry and living leaves, respectively (Figure 6B), plants from the particular accession of *R. hydrolapathum* used in this study can be characterized as extremely potent Cd accumulators, with a root Cd concentration of only 10 mg kg^–1^.

The Cr accumulation potential for a majority of species is usually very low even in contaminated soil. Thus, *Rumex dentatus* accumulated 1.5 and 2.2 mg kg^–1^ Cr in leaves and roots, respectively, when grown in native soil containing 5 mg kg^–1^ Cr [51]. In *Rumex acetosa* from different sites with soil Cr concentrations of 4–63 mg kg^–1^, the Cr concentration in leaves ranged from 0.3 to 2.2 mg kg^–1^ FM [49]. However, as an exception, *Typha angustifolia* plants accumulated 27–29 mg kg^–1^ Cr in shoots [52]. Thus, the Cr accumulation potential in shoots of *R. hydrolapathum* was relatively low, reaching only 7 and 1 mg kg^–1^ in dry and living leaves, respectively (Figure 7B).

In *Rumex acetosa* from different sites with soil Ni concentrations of 46–53 mg kg^–1^, the Ni concentration in leaves ranged from 0.4 to 1.9 mg kg^–1^ FM [49]. For typical Ni excluders, such as *Triticum aestivum*, Ni accumulated up to 11 mg kg^–1^ DM, but Ni indicator species, such as *Trifolium pratense*, accumulated up to 40 mg kg^–1^ Ni [53]. The hyperaccumulation threshold concentration for Ni has been set at 1000 mg kg^–1^, and many endemic species of genus *Alyssum* may significantly exceed this level. For example, *Alyssum murale* plants accumulated 14,772–39,138 mg kg^–1^ Ni in their shoots [53]. In identical conditions, the leaves of the coast-specific dune plant *Alyssum montanum* subsp. *gmelinii* accumulated 626 mg kg^–1^ Ni, and those of *A. murale* accumulated 21,976 mg kg^–1^ [54]. Consequently, the accumulation potential for Ni in the leaves of *R. hydrolapathum* can be characterized as relatively high, as the Ni concentration reached 277 and 131 mg kg^–1^ DM in dry and living leaves, respectively (Figure 8B).

The poor capability of Pb translocation from roots to shoots in a majority of plant species has been associated with the presence of efficient root barriers for apoplastic transport of Pb [55], leading to preferential accumulation of the metal in roots. Only a few plant species have been described as being able to accumulate comparable amounts of Pb in aboveground parts. Among these, *Rumex crispus* plants, grown on mine tailings, accumulated 351–832 and 357–847 mg kg^–1^ Pb in roots and leaves, respectively [15]. *Rumex nepalensis* plants were able to accumulate up to 330 and 1225 mg kg^–1^ Pb in shoots and roots, respectively, when cultivated with intercropping with *Lolium perenne* and *Trifolium repens* [18]. In contrast, non-accumulating species of the genus, such as *Rumex dentatus*, accumulated only 1.0 and 6.5 mg kg^–1^ Pb in stems and roots, respectively, when grown in soil containing 15 mg kg^–1^ Pb [51]. The shoot accumulation potential for Pb reached only 29 mg kg^–1^ for the pseudo-metallophyte species *Rumex acetosa* [19]. In *Rumex acetosa* from different sites with soil Pb concentrations of 40–286 mg kg^–1^, the Pb concentration in leaves ranged from 0.5 to 3.0 mg kg^–1^ [49]. It seems that the accumulation potential of Pb was relatively high for *R. hydrolapathum* compared to other *Rumex* species, as the Pb concentration in dry leaves reached 307–775 mg kg^–1^, but the roots outside the soil accumulated 3325–6050 mg kg^–1^ Pb (Figure 9C,D).

In contrast to other metals, hyperaccumulation of Na^+^ has not been so much formalized. However, the hyperaccumulation threshold concentration of Na^+^ for coastal plant species has been proposed to be in a range of 18–30 g kg^–1^ [56]. In the present study, the level of soil moisture significantly affected Na^+^ accumulation in leaves of *R. hydrolapathum*, with the highest concentration achieved in flooded conditions (Figure 2B). The accumulation potential for Na^+^, up to 30 g kg^–1^ DM in living leaves of *R. hydrolapathum*, indicated that the plant is a Na^+^ hyperaccumulators. In natural conditions, *R. hydrolapathum* belongs to the tight EC-controlling species, regulating tissue electrolyte levels by concomitant changes in Na^+^ and K^+^ concentrations [56]. This was also reflected in the present study, as tissue EC levels showed a saturable response with increasing salinity level (Figure 5A).

The ability to accumulate metals in older leaves with subsequent induction of their senescence was an important adaptive trait for adaptation of *R. hydrolapathum* plants to adverse conditions, including tolerance to salinity and heavy metals. This type of response was previously found for *R. hydrolapathum* plants cultivated under increasing doses of the biogenous heavy metals Mn and Zn [24].

## 5. Conclusions

*R. hydrolapathum* is a perennial dock species with relatively fast growth and high biomass accumulation potential. The present experiments established *R. hydrolapathum* plants as typical metal-tolerant accumulators that are able to concentrate metals in leaf tissues, according to the concept of “shoot accumulator” species [57]. Two features of metal accumulation in *R. hydrolapathum* plants related to organ-level metal compartmentalization are noteworthy. First, dry (dead) leaves had the highest potential for accumulation of Na^+^, K^+^, Cd, and Ni, and it was also high for Cr and Pb. This indicates that metal accumulation in the older leaves with further initiation of the senescence program represents an important adaptation mechanism of the species, and this is the basis of the high tolerance to adverse environmental conditions. Second, high soil moisture induced formation and rapid elongation of roots that were located outside the soil in the surrounding water environment and had a pronounced potential for metal accumulation.

Tolerance to rootzone hypoxia is an especially important feature of plants to be potentially used in different constructed wetland systems for wastewater treatment. *R. hydrolapathum* plants showed optimum growth in waterlogged soil and even soil flooding only had a minor negative effect on plants. Consequently, the plants can be used for the practical purposes of phytoremediation in flooded soils or partially submerged conditions.

## Figures and Tables

**Table 1:** Conditions of the experiments performed with *Rumex hydrolapathum* plants.

Experiment	Soil Moisture	Treatment	Fertilization
Soil Moisture	30%	–	Before treatment: Kristalon Green Label (0.5 g L^–1^), 50 mL per plant
	30%	3 × 100 mL 200 mM NaCl	
	50%	–	
	50%	3 × 100 mL 200 mM NaCl	
	Waterlogged	–	
	Waterlogged	3 × 100 mL 200 mM NaCl	
	Flooded	–	
	Flooded	3 × 100 mL 200 mM NaCl	
Waterlogging with NaCl	Waterlogged	Stepwise increase to 0, 25, 50, 100, 200, 400 mM NaCl in water used for waterlogging	Before treatment: Kristalon Green Label (0.5 g L^–1^), 50 mL per plant
Increasing Soil Cd	60–70%	Stepwise increase to 0, 2, 5, 10, 20, 100 mg Cd L^–1^ of soil in a form of solubilized CdSO_4_ after the final transplanting of seedlings	Kristalon Green Label (0.5 g L^–1^), 50 mL per plant, once every two weeks
Increasing Soil Cr	60–70%	Stepwise increase to 0, 2, 5, 10, 20, 100 mg Cr L^–1^ of soil in the form of solubilized K_2_Cr_2_O_7_ after the final transplanting of seedlings	Kristalon Green Label (0.5 g L^–1^), 50 mL per plant, once every two weeks
Increasing Soil Ni	60–70%	Ni 0, 50, 100, 200, 500, 1000 mg L^–1^ of soil in the dry form of NiSO_4_ before the final transplanting of seedlings	Kristalon Green Label (0.5 g L^–1^), 50 mL per plant, once every two weeks
Increasing Soil Pb Nitrate and Pb Acetate	60–70%	Stepwise increase to 0, 200, 500, 1000 mg Pb L^–1^ of soil in the form of solubilized Pb(NO_3_)_2_ or Pb(CH_3_COO)_2_ after the final transplanting of seedlings	Kristalon Green Label (0.5 g L^–1^), weeks 50 mL per plant, once every two

## Data Availability

All data reported here is available from the authors upon request.

## Supplementary Materials

The following supporting information can be downloaded at: https://www.mdpi.com/article/10.3390/life13071604/s1, Figure S1: *Rumex hydrolapathum* plants in salt-affected coastal wetlands; Figure S2: Changes in soil moisture (A) and electrical conductivity (B) in soil of *Rumex hydrolapathum* plants under different moisture treatments and addition of NaCl; Figure S3: Typical *Rumex hydrolapathum* plants in soil moisture experiment two weeks after the start of the treatment; Figure S4: Relative effect of soil moisture on time course of chlorophyll concentration (A), relative effect of NaCl treatment on time course of chlorophyll concentration using different soil moisture regimes (B), relative effect of soil moisture on time course of Performance Index (C), relative effect of NaCl treatment on time course of Performance Index using different soil moisture regimes (D) of *Rumex hydrolapathum* plants; Figure S5: Effect of NaCl gradient on soil extract electrical conductivity (A) and soil Na^+^ concentration (B) in containers with *Rumex hydrolapathum* plants at the termination of the experiment; Figure S6: Typical *Rumex hydrolapathum* plants in NaCl concentration gradient experiment two weeks (above) and five weeks (below) after the start of the treatment in waterlogged conditions; Figure S7: Relative effect of increasing NaCl concentration on time course of chlorophyll concentration (A) and time course of Performance Index (B) of *Rumex hydrolapathum* plants; Figure S8: Typical *Rumex hydrolapathum* plants in Cd treatment experiment seven weeks after the start of the treatment; Figure S9: Typical *Rumex hydrolapathum* plants in Cr treatment experiment four weeks after the start of the treatment; Figure S10: Typical *Rumex hydrolapathum* plants in Ni treatment experiment seven weeks after the start of the treatment; Figure S11: Typical *Rumex hydrolapathum* plants in Pb treatment experiment four weeks after the start of the treatment; Figure S12: Effect of increasing concentration of Pb nitrate and Pb acetate on soil pH in containers with *Rumex hydrolapathum* plants at the termination of the experiment; Figure S13: Effect of increasing concentration of Pb nitrate and Pb acetate on chlorophyll concentration at Week 2 (A) and Week 7 (B) and on Performance Index at Week 2 (C) and Week 5 (D) in leaves of *Rumex hydrolapathum* plants.

## References

[1] Marín-Muñiz J.L., Zitácuaro-Contreras I., Ortega-Pineda G., Álvarez-Hernández L.M., Martínez-Aguilar K.E., López-Roldán A., Zamora S. (2023). Bibliometric analysis of constructed wetlands with ornamental flowering plants: The importance of green technology. Processes.

[2] Mocek-Płóciniak A., Mencel J., Zakrzewski W., Roszkowski S. (2023). Phytoremediation as an effective remedy for removing trace elements from ecosystems. Plants.

[3] Priya A.K., Muruganandam M., Ali S.S., Kornaros M. (2023). Clean-up of heavy metals from contaminated soil by phytoremediation: A multidisciplinary and eco-friendly approach. Toxics.

[4] Moray C., Goolsbay E.W., Bromham L. (2016). The phylogenetic association between salt tolerance and heavy metal hyperaccumulation in Angiosperms. Evol. Biol..

[5] Sruthi P., Shackira A.M., Puthur J.T. (2017). Heavy metal detoxification mechanisms in halophytes: An overview. Wetlands Ecol. Manag..

[6] Waly M.M., Ahmed T., Abunada Z., Miskovski S.B., Thomson C. (2022). Constructed wetland for sustainable and low-cost wastewater treatment: Review article. Land.

[7] Agaton C.B., Guila P.M.C. (2023). Ecosystem services valuation of constructed wetlands as a nature-based solution to wastewater treatment. Earth.

[8] Sager L., Clerc C., Caffrey J.M., Dutartre A., Haury J., Murphy K.J., Wade P.M. (2006). Factors influencing the distribution of *Hydrocharis morsus-ranae* L. and *Rumex hydrolapathum* Huds. in a mowed low-lying marshland, Réserve de Cheyres, lac de Neuchâtel, Switzerland. Macrophytes in Aquatic Ecosystems: From Biology to Management.

[9] Visser E.J.W., Blom C.W.P.M., Voesenek L.A.C.J. (1996). Flooding-induced adventitious rooting in *Rumex*: Morphology and development in an ecological perspective. Acta Bot. Neerl..

[10] Grevilliot F., Muller S. (2002). Grassland ecotopes of the upper Meuse as references for habitats and biodiversity restoration: A synthesis. Landsc. Ecol..

[11] Grevilliot F., Krebs L., Muller S. (1998). Comparative importance and interference of hydrological conditions and soil nutrient gradients in floristic biodiversity in flood meadows. Biodiv. Conserv..

[12] Voesenek L.A.C.J., Benschop J.J., Bou J., Cox M.C.H., Groeneveld H.W., Millenaar F.F., Vreeburg R.A.M., Peeters A.J.M. (2003). Interactions between plant hormones regulate submergence-induced shoot elongation in the flooding-tolerant dicot *Rumex palustris*. Ann. Bot..

[13] Tang S., Fang Y. (2001). Copper accumulation by *Polygonum microcephalum* D. Don and *Rumex hastatus* D. Don from copper mine spoils in Yunnan Province, P.R. China. Environ. Geol..

[14] Zhuang P., Wang Q.W., Wang H.B., Shu W.S. (2007). Phytoextraction of heavy metals by eight plant species in field. Water Air Soil Pollut..

[15] Xue X., Liu G. (2014). Resistance and distribution to heavy metals of Zoysia sinica Hance and Rumex crispus. Adv. Mater. Res..

[16] Vondráková S., Száková J., Drábek O., Tejnecký V., Hejcman M., Müllerová V., Tlustoð P. (2015). Aluminium uptake and translocation in Al hyperaccumulator *Rumex obtusifolius* is affected by low-molecular weight organic acids content and soil pH. PLoS ONE.

[17] Zhao Y.-H., Jing J.-W., Wang X.-T., Yue H.-M., Niu X.-Y., Fang J.-P. (2016). Study on heavy metals bioaccumulation characteristics and tolerance to pioneer plants from Central Tibet mining area. Acta Agrestia Sin..

[18] Wen W., Zhao H., Ma J., Li Z., Li H., Zhu X., Shao J., Yang Z., Yang Y., He F. (2018). Effects of mutual intercropping on Pb and Zn accumulation of accumulator plants *Rumex nepalensis*, *Lolium perenne* and *Trifolium repens*. Chem. Ecol..

[19] Barrutia O., Epelde L., García-Plazaola J.I., Garbisu C., Becerril J.M. (2009). Phytoextraction potential of two *Rumex acetosa* L. accessions collected from metalliferous and non-metalliferous sites: Effect of fertilization. Chemosphere.

[20] Ye M., Liao B., Li J.T., Mengoni A., Hu M., Luo W.C., Shu W.S. (2012). Contrasting patterns of genetic divergence in two sympatric pseudo-metallophytes: *Rumex acetosa* L. and *Commelina communis* L.. BMC Evol. Biol..

[21] Čiamporová M., Nadubinská M., Banásová V., Ďurišová E., Zelinová V., Horak O., Gruber D., Lichtscheidl I.K. (2021). Structural traits of leaf epidermis correspond to metal tolerance in *Rumex acetosella* populations growing on metal-contaminated soils. Protoplasma.

[22] Jutila H. (2001). How does grazing by cattle modify the vegetation of coastal grasslands along the Baltic Sea?. Ann. Bot. Fenn..

[23] Tyler T., Herbertsson L., Olofsson J., Olsson P.A. (2021). Ecological indicator and traits values for Swedish vascular plants. Ecol. Indic..

[24] Ievinsh G., Dišlere E., Karlsons A., Osvalde A., Vikmane M. (2020). Physiological responses of wetland species *Rumex hydrolapathum* to increased concentration of biogenous heavy metals Zn and Mn in substrate. Proc. Latv. Acad. Sci. B.

[25] Landorfa-Svalbe Z., Andersone-Ozola U., Ievinsh G. (2023). Type of anion largely determines salinity tolerance in four *Rumex* species. Plants.

[26] Snowden R.E.D., Wheeler B.D. (1993). Iron toxicity to fen plant species. J. Ecol..

[27] Strasser R.J., Srivastava A., Tsimilli-Michael M., Yunus M., Pathre U., Mohanty P. (2000). The fluorescence transient as a tool to characterise and screen photosynthetic samples. Probing Photosynthesis: Mechanisms, Regulation and Adaptation.

[28] Gailite A., Andersone-Ozola U., Samsone I., Karlsons A., Ievinsh G. (2023). Ecophysiology of endangered plant species *Saussurea esthonica*: Effect of mineral nutrient availability and soil moisture. Plants.

[29] Schat H. (1984). A comparative ecophysiological study on the effects of waterlogging and submergence on dune slack plants: Growth, survival and mineral nutrition in sand culture experiments. Oecologia.

[30] Fukao T., Barrera-Figueroa B.E., Juntawong P., Peña-Castro J.M. (2019). Submergence and waterlogging stress in plants: A review highlighting research opportunities and understudied aspects. Front. Plant Sci..

[31] Samsone I., Ievinsh G. (2018). Different plant species accumulate various concentration of Na^+^ in a sea-affected coastal wetland during a vegetation season. Environ. Exp. Biol..

[32] Shani U., Ben-Gal A., Tripler E., Dudley L.M. (2007). Plant response to the soil environment: An analytical model integrating yield, water, soil type, and salinity. Water Resour. Res..

[33] Kalaji H.M., Jajoo A., Oukarroum A., Brestic M., Zivcak M., Samborska I.A., Cetner M.D., Łukasik I., Goltsev V., Ladle R.J. (2016). Chlorophyll a fluorescence as a tool to monitor physiological status of plants under abiotic stress conditions. Acta Physiol. Plant..

[34] Hniličková H., Hnilička F., Martinková J., Kraus K. (2017). Effects of salt stress on water status, photosynthesis and chlorophyll fluorescence of rocket. Plant Soil Environ..

[35] Huang C., Wei G., Jie Y., Wang L., Zhou H., Ran C., Anjum S.A. (2014). Effects of concentrations of sodium chloride on photosynthesis, antioxidative enzymes, growth and fiber yield of hybrid ramie. Plant Physiol. Biochem..

[36] Najar R., Aydi S., Sassi-Aydi S., Zarai A., Abdelly C. (2019). Effect of salt stress on photosynthesis and chlorophyll fluorescence in *Medicago truncatula*. Plant Biosyst..

[37] Qiu N., Lu Q., Lu C. (2003). Photosynthesis, photosystem II efficiency and the xanthophyll cycle in the salt-adapted halophyte *Atriplex centralasiatica*. New Phytol..

[38] Redondo-Gómez S., Mateos-Naranjo E., Figueroa M.E., Davy A.J. (2010). Salt stimulation of growth and photosynthesis in an extreme halophyte, *Arthrocnemum macrostachyum*. Plant Biol..

[39] Redondo-Gómez S., Wharmby C., Castillo J.M., Mateos-Naranjo E., Luque C.J., de Cires A., Luque T., Davy A.J., Figueroa M.E. (2006). Growth and photosynthetic responses to salinity in an extreme halophyte, *Sarcocornia fruticosa*. Physiol. Plant..

[40] Moravčiková D., Žiarovská J. (2023). The effect of cadmium on plants in terms of the response of gene expression level and activity. Plants.

[41] Ali S., Mir R.A., Tyagi A., Manzar N., Kashyap A.S., Mushtaq M., Raina A., Park S., Sharma S., Mir Z.A. (2023). Chromium toxicity in plants: Signaling, mitigation, and future perspectives. Plants.

[42] van der Pas L., Ingle R.A. (2019). Towards an understanding of the molecular basis of nickel hyperaccumulation in plants. Plants.

[43] Afzal M.R., Naz M., Wan J., Dai Z., Ullah R., ur Rehman S., Du D. (2023). Insights into the mechanisms involved in lead (Pb) tolerance in invasive plants—The current status of understanding. Plants.

[44] Ievinsh G., Landorfa-Svalbe Z., Andersone-Ozola U., Karlsons A., Osvalde A. (2022). Salinity and heavy metal tolerance, and phytoextraction potential of *Ranunculus sceleratus* plants from a sandy coastal beach. Life.

[45] Wyszkowski M., Radziemska M. (2013). Assessment of tri- and hexavalent chromium phytotoxicity on oats (*Avena sativa* L.) biomass and content of nitrogen compounds. Water Air Soil Pollut..

[46] Ahmad S., Mfarrej M.F.B., E-Esawi M.A., Waseem M., Alatawi A., Nafees M., Saleem M.H., Rizwan M., Yasmeen T., Anayat A. (2022). Chromium-resistant *Staphylococcus aureus* alleviates chromium toxicity by developing synergistic relationships with zinc oxide nanoparticles in wheat. Ecotoxicol. Environ. Safety.

[47] Bera A.K., Kanta-Bokaria A.K., Bokaria K. (1999). Effect of tannery effluent on seed germination, seedling growth and chloroplast pigment content in mungbean (*Vigna radiata*). Environ. Ecol..

[48] Istenič D., Arias C.A., Vollertsen J., Nielsen A.H., Wium-Andersen T., Hvitved-Jacobsen T., Brix H. (2012). Improved urban stormwater treatment and pollutant removal pathways in amended wet detention ponds. J. Environ. Sci. Health A.

[49] Gawęda M. (2009). Heavy metal content in common sorrel plants (*Rumex acetosa* L.) obtained from natural sites in Małopolska province. Pol. J. Environ. Stud..

[50] Reeves R.D., Baker A.J.M., Jaffré T., Erskine P.D., Echevarria G., van der Ent A. (2018). A global database for plants that hyperaccumulate metal and metalloid trace elements. New Phytol..

[51] Ullah R., Hadi F., Ahmad S., Jan A.U., Rongliang Q. (2019). Phytoremediation of lead and chromium contaminated soil improves with the endogenous phenolics and proline production in *Parthenium*, *Cannabis*, *Euphorbia*, and *Rumex* species. Water Air Soil Pollut..

[52] Bah A.M., Dai H., Zhao J., Sun H., Cao F., Zhang G., Wu F. (2011). Effects of cadmium, chromium and lead on growth, metal uptake and antioxidative capacity in *Typha angustifolia*. Biol. Trace Elem. Res..

[53] Massoura S.T., Echevarria G., Leclerc-Cessac E., Morel J.L. (2004). Response of excluder, indicator, and hyperaccumulator plants to nickel availability in soils. Austr. J. Soil Res..

[54] Ievinsh G., Andersone-Ozola U., Samsone I. (2020). *Alyssum montanum* subsp. *gmelinii*, a rare plant species from coastal sand dunes, as a potential Ni accumulator: Comparison with *Alyssum murale*. Environ. Exp. Biol..

[55] Zulfiqar U., Farooq M., Hussain S., Maqsood M., Hussain M., Ishfaq M., Ahmad M., Anjum M.Z. (2019). Lead toxicity in plants: Impacts and remediation. J. Environ. Manag..

[56] Ievinsh G., Ieviņa S., Andersone-Ozola U., Samsone I. (2021). Leaf sodium, potassium and electrolyte accumulation capacity of plant species from salt-affected coastal habitats of the Baltic Sea: Towards a definition of Na hyperaccumulation. Flora.

[57] Muszyńska E., Labudda M. (2019). Dual role of metallic trace elements in stress biology—From negative to beneficial impacts on plants. Int. J. Mol. Sci..

