# Supplementary material for: Coastal Wetland Species Rumex hydrolapathum: Tolerance against Flooding, Salinity, and Heavy Metals for Its Potential Use in Phytoremediation and Environmental Restoration Technologies
Source: Life (Basel). 2023 Jul 21;13(7):1604. doi: 10.3390/life13071604 (PMC10381717; doi:10.3390/life13071604)
Supplement: Supplementary file 1 [file life-13-01604-s001.zip › Rumex Life Supplement.pdf]

Coastal Wetland Species *Rumex hydrolapathum*: Tolerance against Flooding, Salinity, and Heavy Metals for its Potential Use in Phytoremediation and Environmental Restoration Technologies

Silvija Ieviņa, Andis Karlsons, Anita Osvalde, Una Andersone-Ozola and Gederts Ievinsh

Supplement

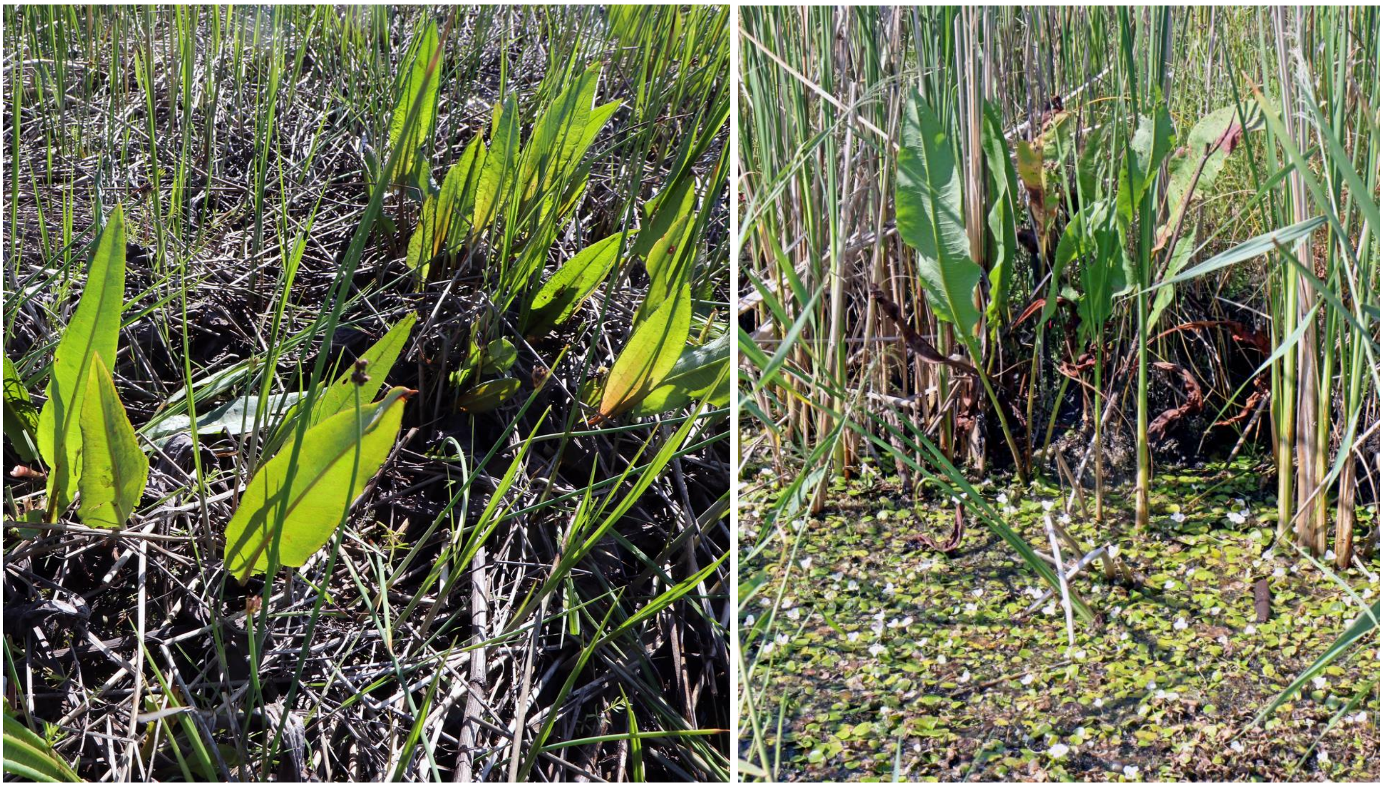

Figure S1. *Rumex hydrolapathum* plants in salt-affected coastal wetlands.

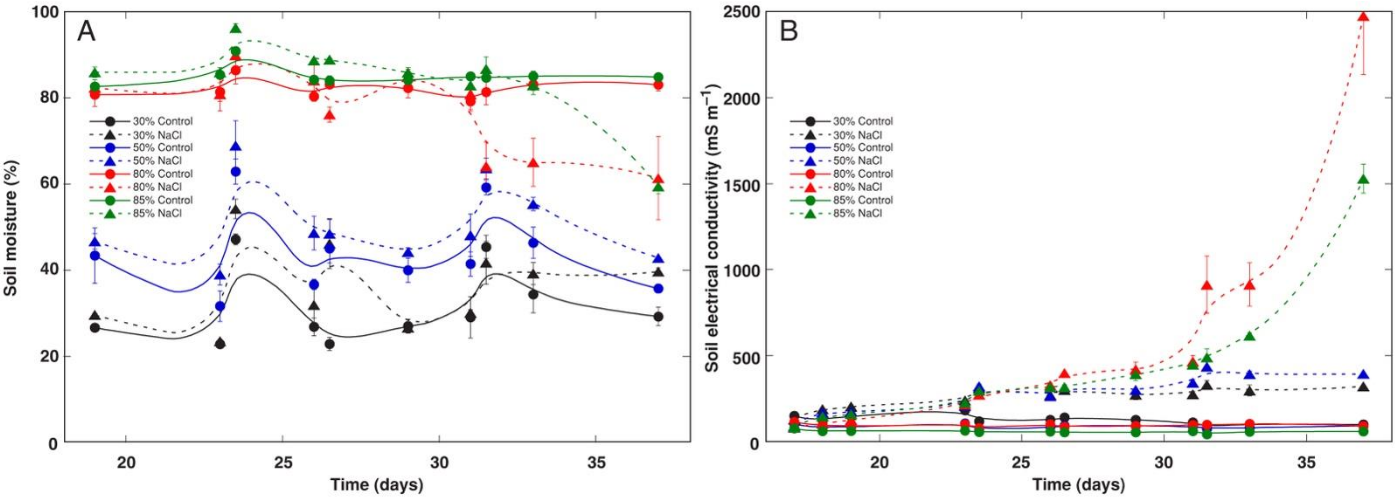

Figure S2. Changes in soil moisture (A) and electrical conductivity (B) in soil of *Rumex hydrolapathum* plants under the effect of different moisture treatment and addition of NaCl. Data are mean values from five replicates ± SE. For each replicate, four independent measurements were performed.

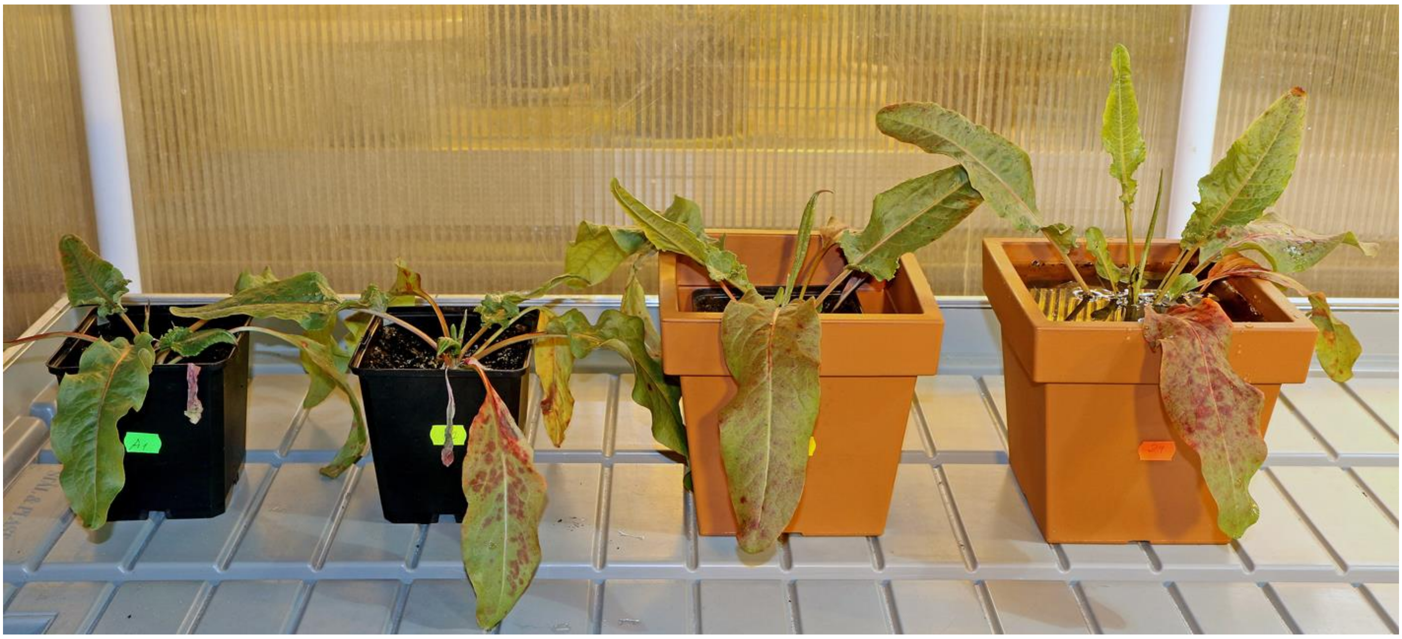

Figure S3. Typical *Rumex hydrolapathum* plants in soil moisture experiment two weeks after the start of the treatment. From left to right: 30% soil moisture, 50% soil moisture, 80% soil moisture (waterlogged soil); 85% soil moisture (flooded soil).

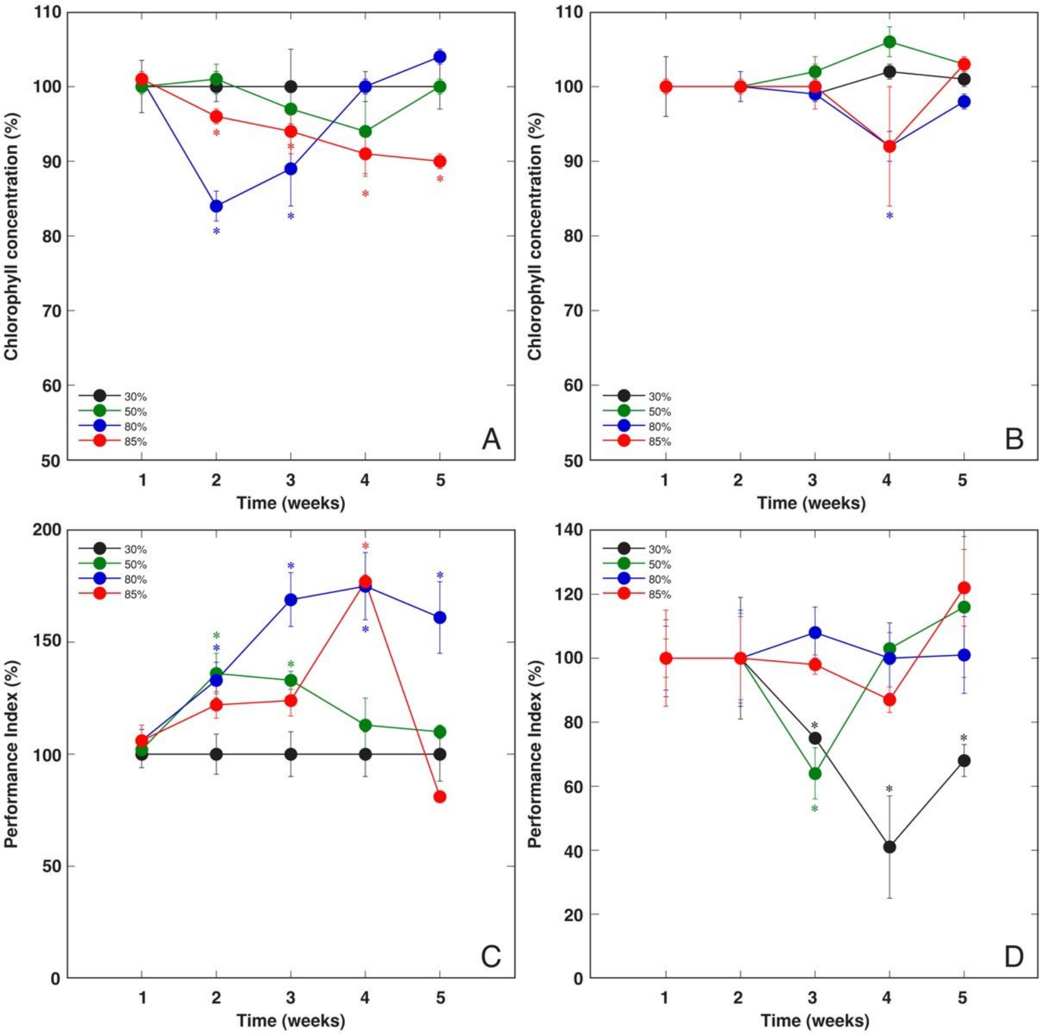

Figure S4. Relative effect of soil moisture on time course of chlorophyll concentration (A), relative effect of NaCl treatment on time course of chlorophyll concentration at different soil moisture regimes (B), relative effect of soil moisture on time course of Performance Index (C), relative effect of NaCl treatment on time course of Performance Index at different soil moisture regimes (D) of *Rumex hydrolapathum* plants. Data are mean values from 10 independent measurements for each point ± SE. Asterisks of respective color indicate statistically significant differences ($p < 0.05$) from control.

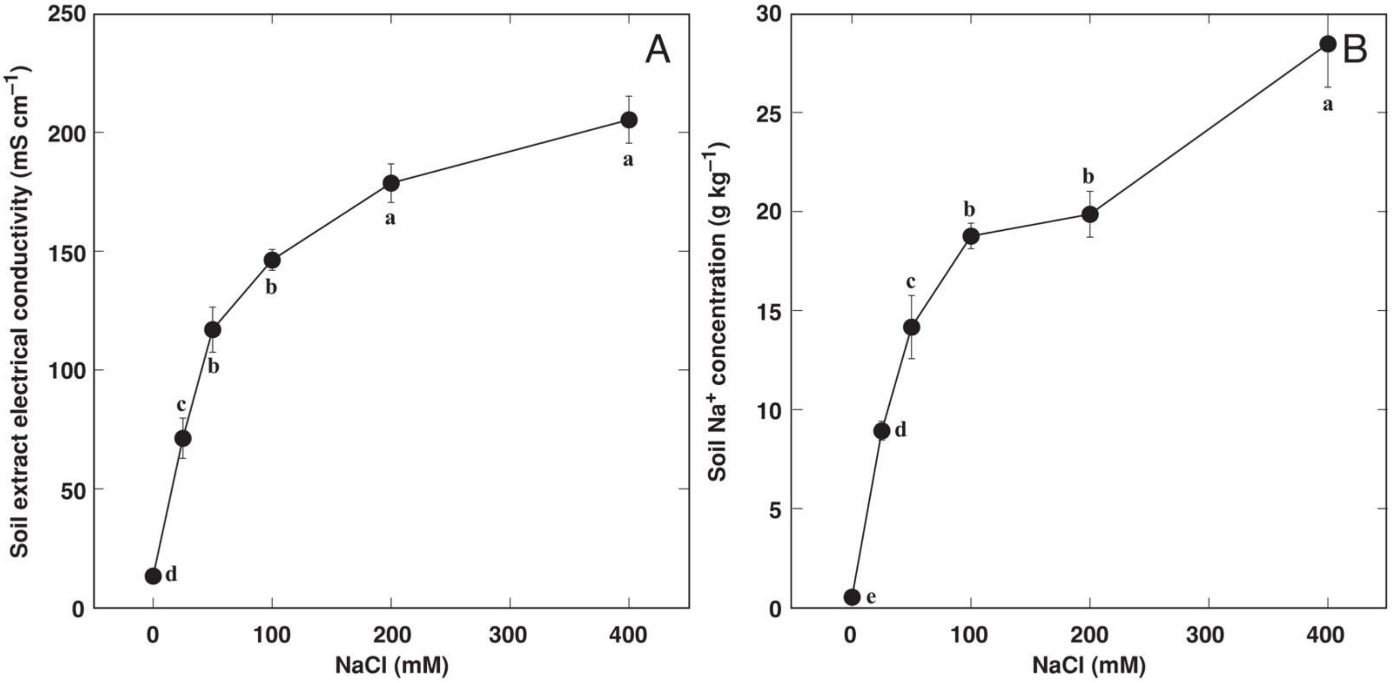

Figure S5. Effect of NaCl gradient on soil extract electrical conductivity (A) and soil Na^+ concentration (B) in containers with *Rumex hydrolapathum* plants at the termination of the experiment. Results represent mean values from five replicates ± SE. Different letters indicate statistically significant differences ($p < 0.05$).

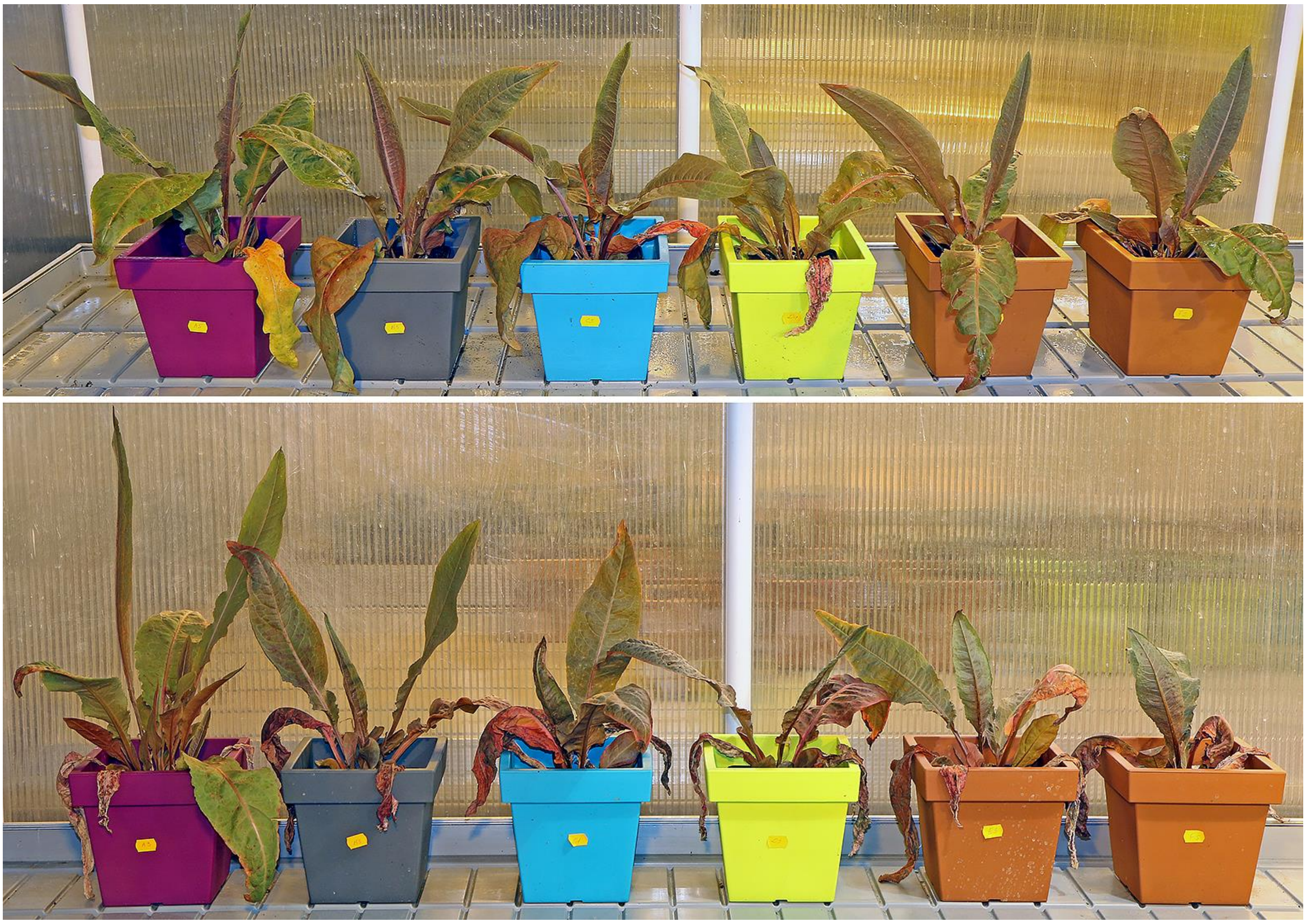

Figure S6. Typical *Rumex hydrolapathum* plants in NaCl concentration gradient experiment two weeks (above) and five weeks (below) after the start of the treatment in waterlogged conditions. From left to right: control, 25 mM NaCl, 50 mM NaCl, 100 mM NaCl, 200 mM NaCl, 400 mM NaCl.

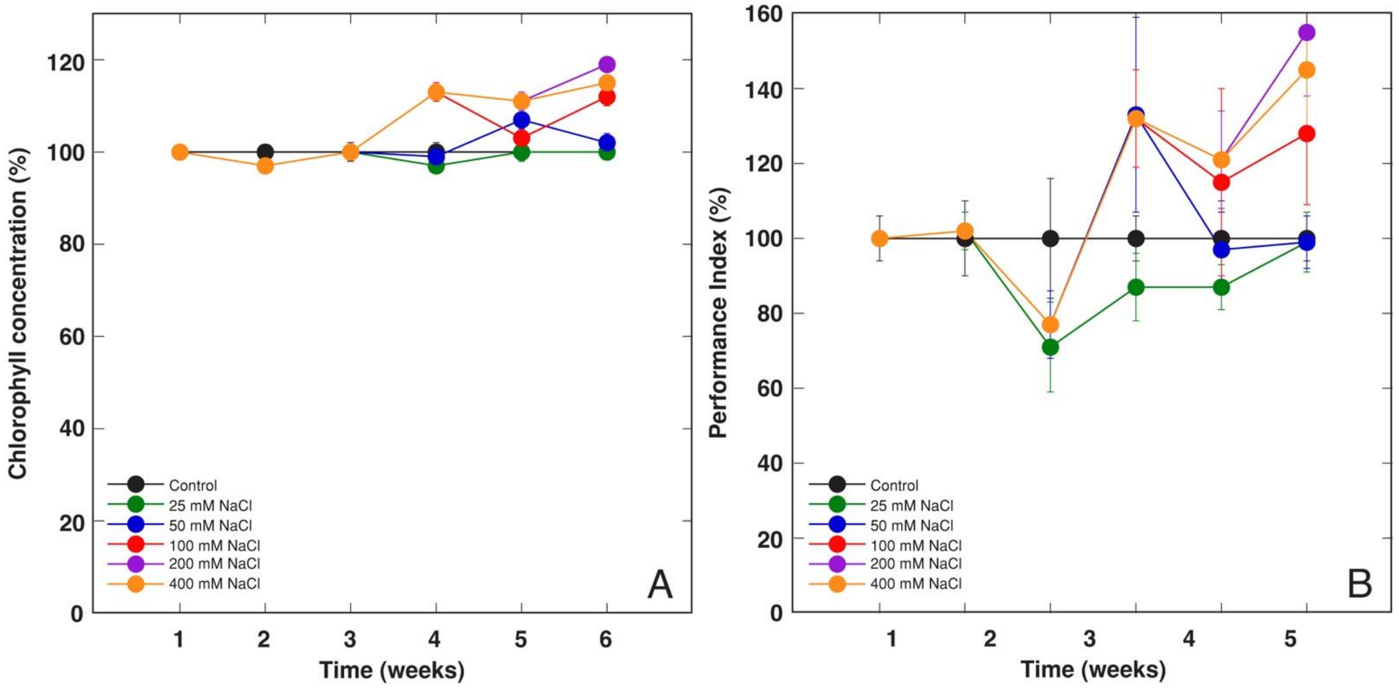

Figure S7. Relative effect of increasing NaCl concentration on time course of chlorophyll concentration (A) and time course of Performance Index (B) of *Rumex hydrolapathum* plants. Results are mean values from 10 independent measurements for each point ± SE.

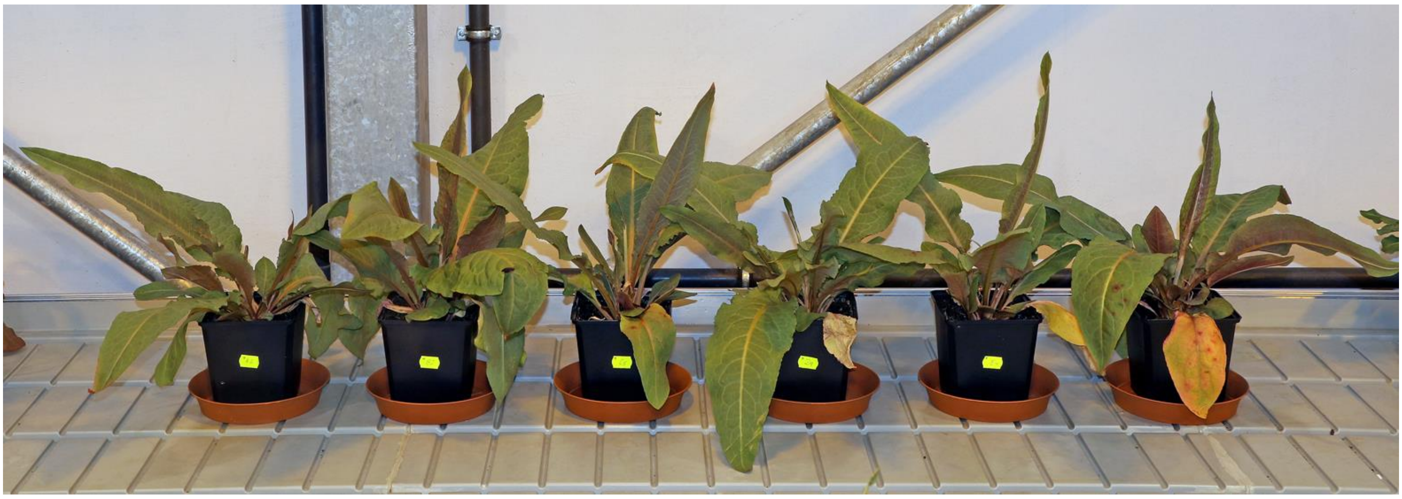

Figure S8. Typical *Rumex hydrolapathum* plants in Cd treatment experiment seven weeks after the start of the treatment. From left to right: control, 2, 5, 10, 20, 50 mg L^{-1} Cd.

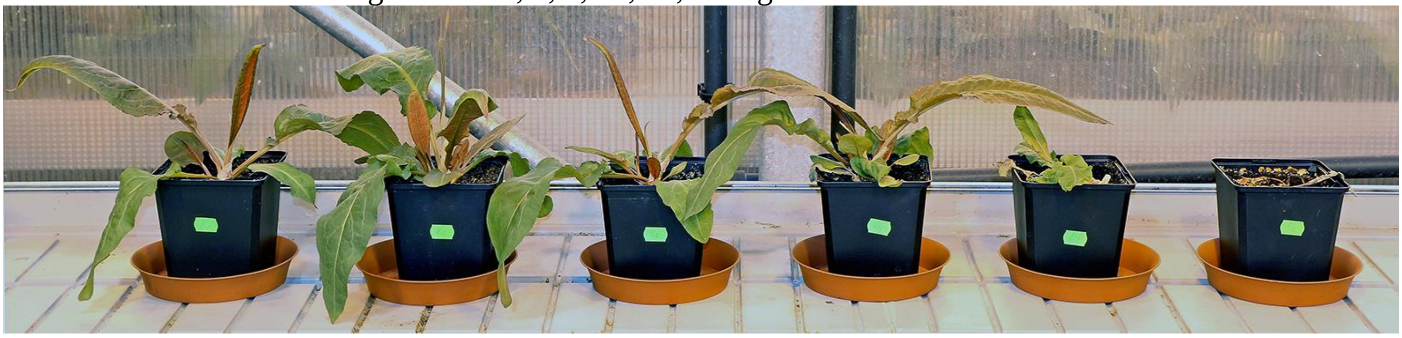

Figure S9. Typical *Rumex hydrolapathum* plants in Cr treatment experiment four weeks after the start of the treatment. From left to right: control, 10, 50, 100, 200, 500 mg L^{-1} Cr.

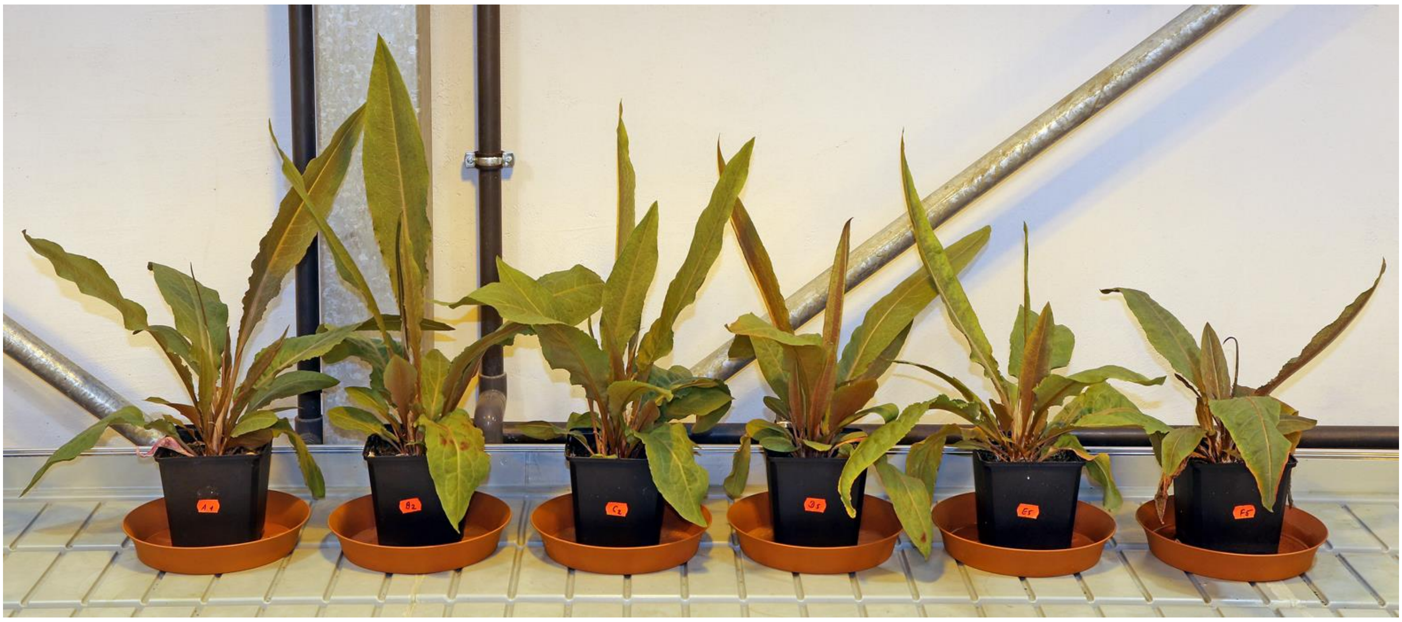

Figure S10. Typical *Rumex hydrolapathum* plants in Ni treatment experiment seven weeks after the start of the treatment. From left to right: control, 50, 100, 200, 500, 1000 mg L^{-1} Ni.

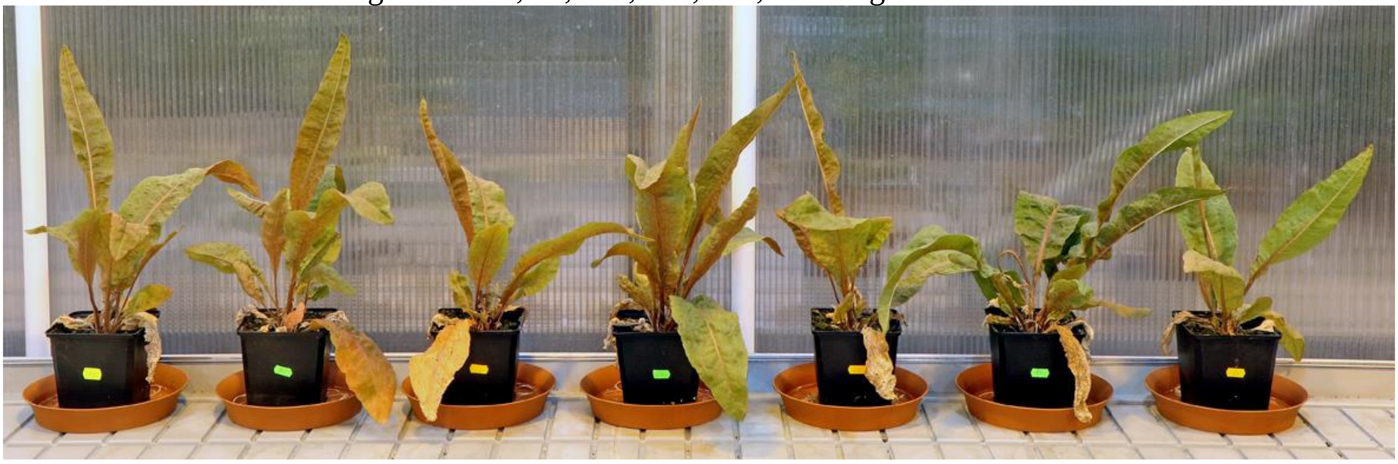

Figure S11. Typical *Rumex hydrolapathum* plants in Pb treatment experiment four weeks after the start of the treatment. From left to right: control, Pb (nitrate) 200 mg L^{-1}, Pb (acetate) 200 mg L^{-1}, Pb (nitrate) 500 mg L^{-1}, Pb (acetate) 500 mg L^{-1}, Pb (nitrate) 1000 mg L^{-1}, Pb (acetate) 1000 mg L^{-1}.

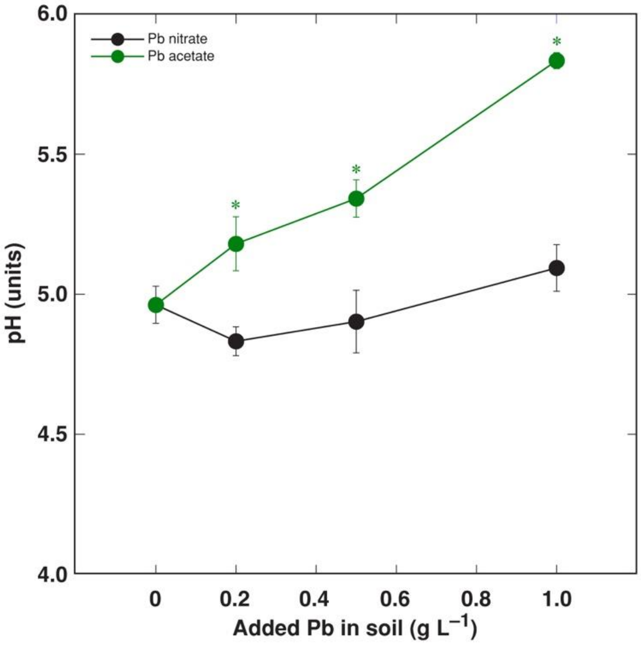

Figure S12. Effect of increasing concentration of Pb nitrate and Pb acetate on soil pH in containers with *Rumex hydrolapathum* plants at the termination of the experiment. Results represent mean values from five replicates ± SE. For each replicate, four independent measurements were performed. Asterisks indicate statistically significant differences ($p < 0.05$) from control.

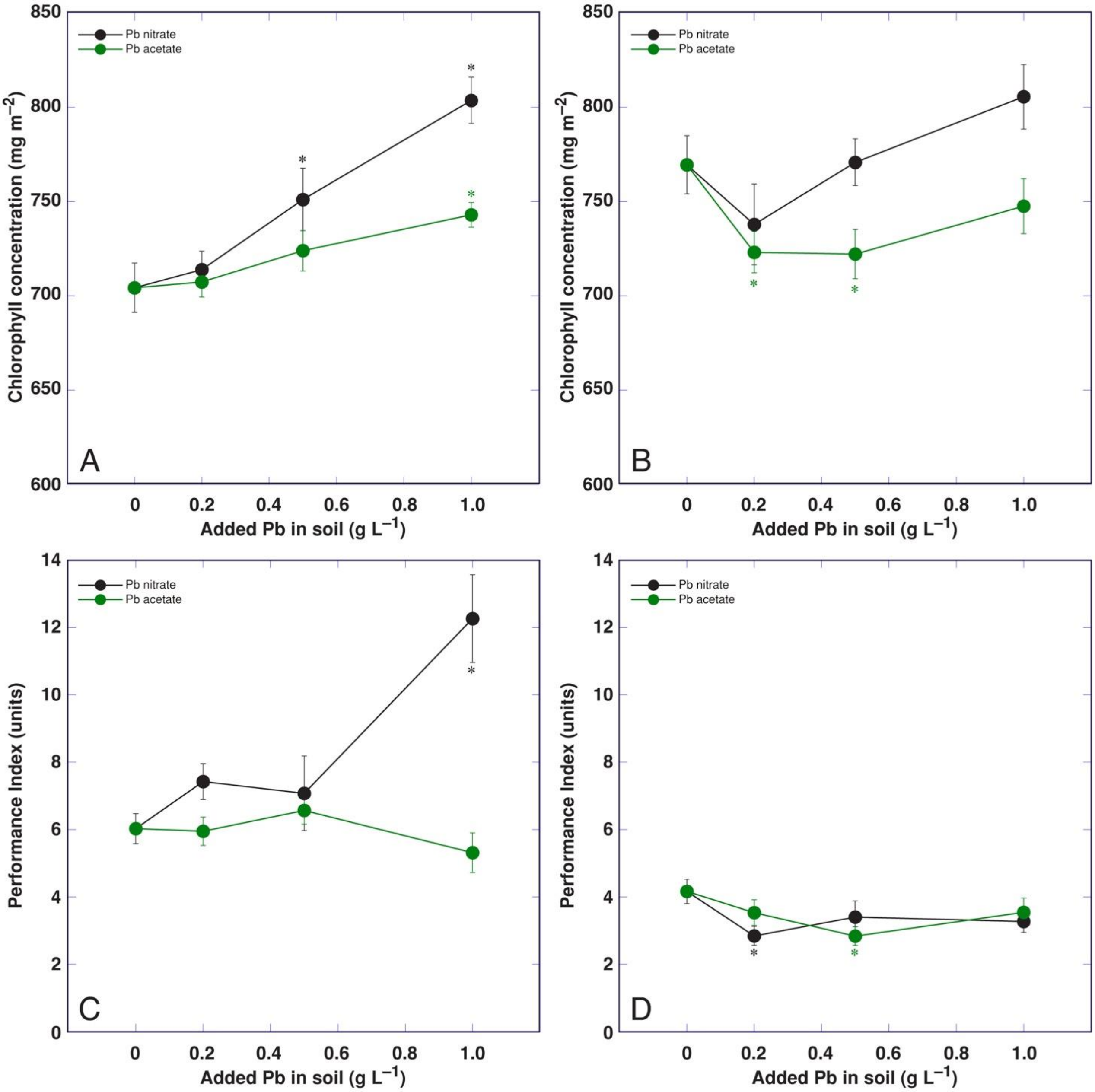

Figure S13. Effect of increasing concentration of Pb nitrate and Pb acetate on chlorophyll concentration on Week 2 (A) and Week 7 (B) and on Performance Index on Week 2 (C) and Week 5 (D) in leaves of *Rumex hydrolapathum* plants. Results represent mean values from five replicates ± SE. Asterisks indicate statistically significant differences ($p < 0.05$) from control.
